# Supplementary material for: Self-supervised learning for generalizable particle picking in cryo-EM micrographs
Source: Cell Rep Methods. 2025 Jul 7;5(7):101089. doi: 10.1016/j.crmeth.2025.101089 (PMC12296464; doi:10.1016/j.crmeth.2025.101089)
Supplement: Document S1. Figures S1–S7 and Tables S1–S6 [file mmc1.pdf]

Cell Reports Methods, Volume *5*

Supplemental information

Self-supervised learning for generalizable particle picking in cryo-EM micrographs

Andreas Zamanos, Panagiotis Koromilas, Giorgos Bouritsas, Panagiotis L. Kastritis, and Yannis Panagakis

Supplemental information

Table S1: **Evaluation metrics for all test datasets, reported for each training dataset. Related to Table 1**. The metrics include: (i) Intersection over Union (IoU), measuring the overlap between ground truth and predicted particles; (ii) Recall, the ratio of correctly predicted particles, prediction counts as a true positive if IoU ≥ 0.6; (iii) Precision, the proportion of retrieved particles that are true positives; and (iv) F1 score, the harmonic mean of recall and precision. For cryo-EMMAE, the difference from the highest-performing or second-highest method is additionally reported for each entry and metric.

Trained on 10291												
	**IoU**			**Recall**			**Precision**			**F1**		
**Test Sets**	Topaz	CrYOLO	EMMAE	Topaz	CrYOLO	EMMAE	Topaz	CrYOLO	EMMAE	Topaz	CrYOLO	EMMAE
10291	0.794	**0.819**	0.579 ↓0.24	**0.955**	0.928	0.597 ↓0.36	0.282	**0.475**	0.434 ↓0.04	0.435	**0.628**	0.503 ↓0.13
10077	0.039	0.566	**0.717** ↑0.15	0.021	0.604	**0.844** ↑0.24	0.219	0.224	**0.464** ↑0.24	0.038	0.327	**0.599** ↑0.27
10590	**0.684**	0.548	0.566 ↓0.12	**0.716**	0.550	0.581 ↓0.14	0.313	0.528	**0.562** ↑0.03	0.436	0.539	**0.571** ↑0.03
10816	0.000	0.010	**0.478** ↑0.47	0.000	0.009	**0.415** ↑0.41	0.000	**0.561**	0.234 ↓0.33	0.000	0.018	**0.299** ↑0.28
10028	0.000	0.558	**0.786** ↑0.23	0.000	0.619	**0.947** ↑0.33	0.000	0.276	**0.658** ↑0.38	0.000	0.382	**0.776** ↑0.39
10081	**0.791**	0.739	0.778 ↓0.01	**0.934**	0.844	0.863 ↓0.07	0.313	**0.673**	0.565 ↓0.11	0.469	**0.749**	0.683 ↓0.07
10096	0.210	0.011	**0.522** ↑0.31	0.135	0.004	**0.436** ↑0.30	0.237	0.161	**0.425** ↑0.19	0.172	0.008	**0.430** ↑0.26
10240	**0.565**	0.445	0.321 ↓0.24	**0.574**	0.428	0.299 ↓0.28	0.389	0.560	**0.563** - 0.00	0.464	**0.485**	0.391 ↓0.09
10406	0.017	0.639	**0.716** ↑0.08	0.008	0.655	**0.814** ↑0.16	0.094	0.187	**0.569** ↑0.38	0.015	0.291	**0.670** ↑0.38
10289	**0.735**	0.649	0.571 ↓0.16	**0.798**	0.664	0.553 ↓0.25	0.208	0.317	**0.361** ↑0.04	0.330	0.429	**0.437** ↑0.01
10737	**0.498**	0.476	0.444 ↓0.05	**0.490**	**0.490**	0.461 ↓0.03	0.205	**0.544**	0.459 ↓0.09	0.289	**0.516**	0.460 ↓0.06
10059	**0.677**	0.465	0.433 ↓0.24	**0.674**	0.436	0.340 ↓0.33	0.334	**0.620**	0.495 ↓0.13	0.447	**0.512**	0.403 ↓0.11
11183	0.489	0.309	**0.533** ↑0.04	0.486	0.293	**0.527** ↑0.04	0.173	**0.323**	0.287 ↓0.04	0.255	0.307	**0.372** ↑0.07
10017	0.454	0.018	**0.498** ↑0.04	0.457	0.006	**0.518** ↑0.06	0.569	0.205	**0.657** ↑0.09	0.507	0.012	**0.579** ↑0.07
Mean	0.425	0.447	**0.567** ↑0.12	0.446	0.467	**0.585** ↑0.12	0.238	0.404	**0.481** ↑0.08	0.276	0.372	**0.512** ↑0.14

Trained on 10077												
	**IoU**			**Recall**			**Precision**			**F1**		
**Test Sets**	Topaz	CrYOLO	EMMAE	Topaz	CrYOLO	EMMAE	Topaz	CrYOLO	EMMAE	Topaz	CrYOLO	EMMAE
10291	**0.747**	0.233	0.601 ↓0.15	**0.851**	0.033	0.620 ↓0.23	0.289	0.057	**0.428** ↑0.14	**0.431**	0.042	0.506 ↑0.08
10077	**0.826**	0.784	0.708 ↓0.12	**0.983**	0.924	0.832 ↓0.15	0.348	**0.506**	0.46 ↓0.05	0.514	**0.654**	0.592 ↓0.06
10590	**0.664**	0.459	0.565 ↓0.01	**0.698**	0.368	0.577 ↓0.12	0.247	0.465	**0.553** ↑0.09	0.365	0.411	**0.565** ↑0.15
10816	0.489	0.000	**0.502** ↑0.01	0.389	0.000	**0.474** ↑0.09	0.147	0.000	**0.302** ↑0.16	**0.213**	0.000	0.369 ↑0.16
10028	**0.836**	0.815	0.788 ↓0.05	**0.995**	0.955	0.950 ↓0.05	0.408	0.630	**0.650** ↑0.02	0.579	0.759	**0.772** ↑0.01
10081	0.680	0.454	**0.779** ↑0.10	0.772	0.329	**0.865** ↑0.09	0.134	0.355	**0.550** ↑0.20	0.228	0.342	**0.672** ↑0.33
10096	0.018	0.025	**0.537** ↑0.51	0.003	0.002	**0.445** ↑0.44	0.010	0.036	**0.416** ↑0.38	0.005	0.004	**0.430** ↑0.43
10240	**0.615**	0.308	0.343 ↓0.27	**0.620**	0.206	0.320 ↓0.30	0.305	0.437	**0.564** ↑0.13	**0.409**	0.280	0.408 - 0.00
10406	0.775	**0.782**	0.717 ↓0.07	0.918	**0.930**	0.818 ↓0.11	0.440	**0.637**	0.575 ↓0.06	0.595	**0.756**	0.675 ↓0.08
10289	**0.667**	0.339	0.576 ↓0.09	**0.683**	0.140	0.559 ↓0.12	0.184	0.160	**0.357** ↑0.17	**0.290**	0.149	0.436 ↑0.15
10737	**0.596**	0.070	0.487 ↓0.11	**0.635**	0.043	0.502 ↓0.13	0.311	0.355	**0.463** ↑0.11	0.418	0.077	**0.482** ↑0.06
10059	**0.645**	0.196	0.432 ↓0.21	**0.612**	0.057	0.338 ↓0.27	0.413	0.191	**0.495** ↑0.08	**0.493**	0.088	0.402 ↓0.09
11183	**0.669**	0.014	0.533 ↓0.14	**0.703**	0.003	0.534 ↓0.17	0.210	0.054	**0.288** ↑0.08	0.323	0.006	**0.374** ↑0.05
10017	0.346	0.035	**0.486** ↑0.14	0.247	0.002	**0.505** ↑0.26	0.171	0.021	**0.645** ↑0.47	0.202	0.004	**0.566** ↑0.36
Mean	**0.612**	0.322	0.575 ↓0.04	**0.651**	0.285	0.596 ↓0.06	0.258	0.279	**0.482** ↑0.14	0.362	0.255	**0.518** ↑0.11

Trained on 10590												
	**IoU**			**Recall**			**Precision**			**F1**		
**Test Sets**	Topaz	CrYOLO	EMMAE	Topaz	CrYOLO	EMMAE	Topaz	CrYOLO	EMMAE	Topaz	CrYOLO	EMMAE
10291	**0.714**	0.665	0.484 ↓0.23	**0.784**	0.717	0.502 ↓0.28	0.285	0.414	**0.449** ↑0.04	0.418	**0.525**	0.474 ↓0.05
10077	0.098	**0.690**	0.407 ↓0.28	0.116	**0.785**	0.477 ↓0.31	**0.644**	0.235	0.458 ↓0.19	0.197	0.362	**0.467** ↑0.11
10590	**0.788**	0.742	0.588 ↓0.20	**0.915**	0.834	0.599 ↓0.32	0.284	0.500	**0.543** ↑0.04	0.433	**0.625**	0.570 ↓0.06
10816	0.020	0.061	**0.127** ↑0.07	0.006	**0.059**	0.037 ↓0.02	0.023	**0.648**	0.036 ↓0.61	0.010	**0.108**	0.036 ↓0.07
10028	0.028	0.716	**0.787** ↑0.07	0.026	0.868	**0.939** ↑0.07	**0.714**	0.289	0.626 ↓0.09	0.050	0.434	**0.751** ↑0.32
10081	**0.829**	0.797	0.784 ↓0.04	**0.958**	0.921	0.870 ↓0.09	0.165	0.343	**0.551** ↑0.21	0.282	0.500	**0.675** ↑0.18
10096	**0.604**	0.326	0.533 ↓0.07	**0.543**	0.104	0.440 ↓0.10	0.171	0.131	**0.439** ↑0.27	0.260	0.116	**0.439** ↑0.18
10240	**0.740**	0.672	0.319 ↓0.42	**0.801**	0.701	0.297 ↓0.50	0.294	0.476	**0.55** ↑0.07	0.430	**0.567**	0.386 ↓0.18
10406	0.369	0.667	**0.717** ↑0.05	0.404	0.689	**0.813** ↑0.12	**0.568**	0.271	0.557 ↓0.01	0.472	0.389	**0.661** ↑0.19
10289	**0.705**	0.700	0.527 ↓0.18	0.742	**0.743**	0.509 ↓0.23	0.230	0.294	**0.351** ↑0.06	0.351	**0.421**	0.415 ↓0.01
10737	**0.539**	0.400	0.472 ↓0.07	**0.562**	0.415	0.485 ↓0.08	0.352	**0.549**	0.455 ↑0.09	0.433	**0.473**	0.470 - 0.00
10059	0.488	**0.530**	0.214 ↓0.32	0.430	**0.456**	0.170 ↓0.29	**0.459**	0.531	**0.513** ↓0.02	0.444	**0.491**	0.255 ↓0.24
11183	0.044	**0.276**	0.115 ↓0.16	0.034	**0.239**	0.043 ↓0.20	0.082	**0.314**	0.072 ↓0.24	0.048	**0.271**	0.054 ↓0.22
10017	**0.762**	0.468	0.506 ↓0.26	**0.847**	0.278	0.521 ↓0.33	0.242	0.274	**0.626** ↑0.35	0.376	0.276	**0.569** ↑0.19
Mean	0.481	**0.551**	0.470 ↓0.08	0.512	**0.558**	0.479 ↓0.08	0.322	0.376	**0.444** ↑0.07	0.300	0.397	**0.444** ↑0.05

Trained on 10816												
	**IoU**			**Recall**			**Precision**			**F1**		
**Test Sets**	Topaz	CrYOLO	EMMAE	Topaz	CrYOLO	EMMAE	Topaz	CrYOLO	EMMAE	Topaz	CrYOLO	EMMAE
10291	0.498	**0.732**	0.571 ↓0.16	0.231	**0.799**	0.584 ↓0.22	0.040	0.306	**0.427** ↑0.12	0.068	0.443	**0.493** ↑0.05
10077	0.587	0.629	**0.712** ↑0.08	0.435	0.622	**0.833** ↑0.21	0.050	0.102	**0.455** ↑0.35	0.090	0.175	**0.589** ↑0.41
10590	0.452	**0.729**	0.577 ↓0.15	0.170	**0.795**	0.588 ↓0.21	0.026	0.272	**0.548** ↑0.28	0.045	0.405	**0.567** ↑0.16
10816	**0.798**	0.734	0.511 ↓0.29	**0.931**	0.800	0.511 ↓0.42	0.134	0.363	**0.443** ↑0.08	0.234	**0.499**	0.475 ↓0.02
10028	0.517	0.660	**0.781** ↑0.12	0.289	0.735	**0.943** ↑0.21	0.030	0.193	**0.642** ↑0.45	0.054	0.306	**0.764** ↑0.46
10081	0.426	**0.801**	0.747 ↓0.05	0.139	**0.909**	0.828 ↓0.08	0.011	0.211	**0.554** ↑0.34	0.020	0.342	**0.664** ↑0.32
10096	0.358	0.378	**0.495** ↑0.12	0.128	0.205	**0.406** ↑0.20	0.029	0.221	**0.461** ↑0.24	0.047	0.213	**0.432** ↑0.22
10240	0.500	**0.683**	0.338 ↓0.35	0.269	**0.708**	0.311 ↓0.40	0.058	0.353	**0.545** ↑0.19	0.095	**0.471**	0.396 ↓0.08
10406	0.513	0.586	**0.701** ↑0.12	0.216	0.455	**0.792** ↑0.34	0.031	0.125	**0.557** ↑0.43	0.054	0.196	**0.654** ↑0.46
10289	0.449	**0.724**	0.572 ↓0.15	0.205	**0.765**	0.554 ↓0.21	0.038	0.216	**0.354** ↑0.14	0.064	0.337	**0.432** ↑0.10
10737	0.612	**0.733**	0.351 ↓0.38	0.565	**0.791**	0.364 ↓0.43	0.078	0.233	**0.453** ↑0.22	0.137	0.360	**0.404** ↑0.04
10059	0.491	**0.602**	0.416 ↓0.19	0.222	**0.532**	0.324 ↓0.21	0.087	0.415	**0.496** ↑0.08	0.125	**0.466**	0.392 ↓0.07
11183	0.526	**0.614**	0.493 ↓0.12	0.397	**0.609**	0.482 ↓0.13	0.080	0.204	**0.297** ↑0.09	0.133	0.306	**0.368** ↑0.06
10017	0.488	0.409	**0.489** - 0.00	0.280	0.300	**0.508** ↑0.21	0.054	0.344	**0.649** ↑0.31	0.091	0.320	**0.570** ↑0.25
Mean	0.515	**0.644**	0.554 ↓0.09	0.320	**0.645**	0.573 ↓0.07	0.053	0.254	**0.492** ↑0.24	0.090	0.346	**0.514** ↑0.17

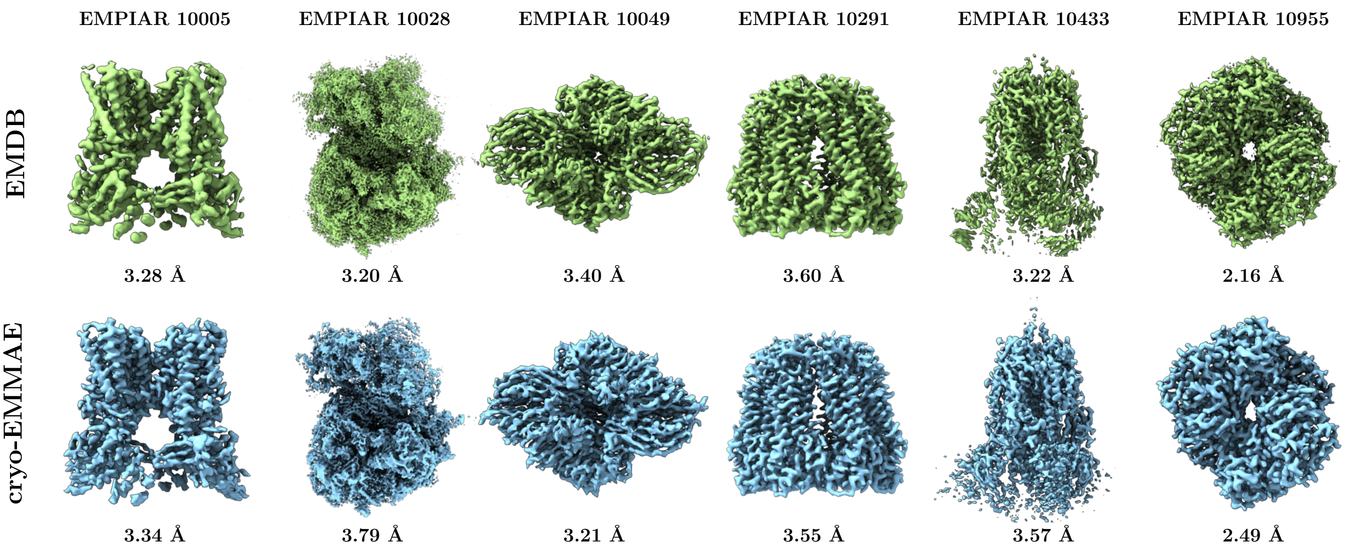

Figure S1: **Visualization of 3D reconstructed density maps from the published volumes in EMDB and cryo-EMMAE across six different EMPIAR datasets. Related to Real Case Studies subsection of the main manuscript.** EMDB maps are shown in green, while those generated using cryo-EMMAE are shown in blue. Resolutions are also reported under each volume. Maps produced with cryo-EMMAE underwent only one round of 2D classification and were not subjected to any post-processing of the 3D reconstructed map.

Table S2: **Comparison of 3D reconstruction resolutions and the number of particles used between the EMPIAR published datasets and the cryo-EMMAE particle picker. Related to Real Case Studies subsection of the main manuscript.** Results are reported after one round of 2D classification and selection, with mean values also provided.

Symmetry	EMPIAR	3D Reconstruction Resolution		Number of Particles	
		EMPIAR	cryo-EMMAE	EMPIAR	cryo-EMMAE
C4	10005	**3.28Å**	3.34Å ↑0.06Å	35,645	44,215
C1	10028	**3.20Å**	3.79Å ↑0.59Å	105,247	134,330
C2	10049	3.40Å	**3.21Å** ↓0.19Å	108,544	138,153
C8	10291	3.60Å	**3.55Å** ↓0.05Å	54,536	49,975
C3	10433	**3.22Å**	3.45Å ↑0.23Å	54,395	55,733
D2	10955	**2.16Å**	2.49Å ↑0.33Å	88,731	40,418
	Mean	**3.14Å**	3.30Å ↑0.16Å	74,516	77,137

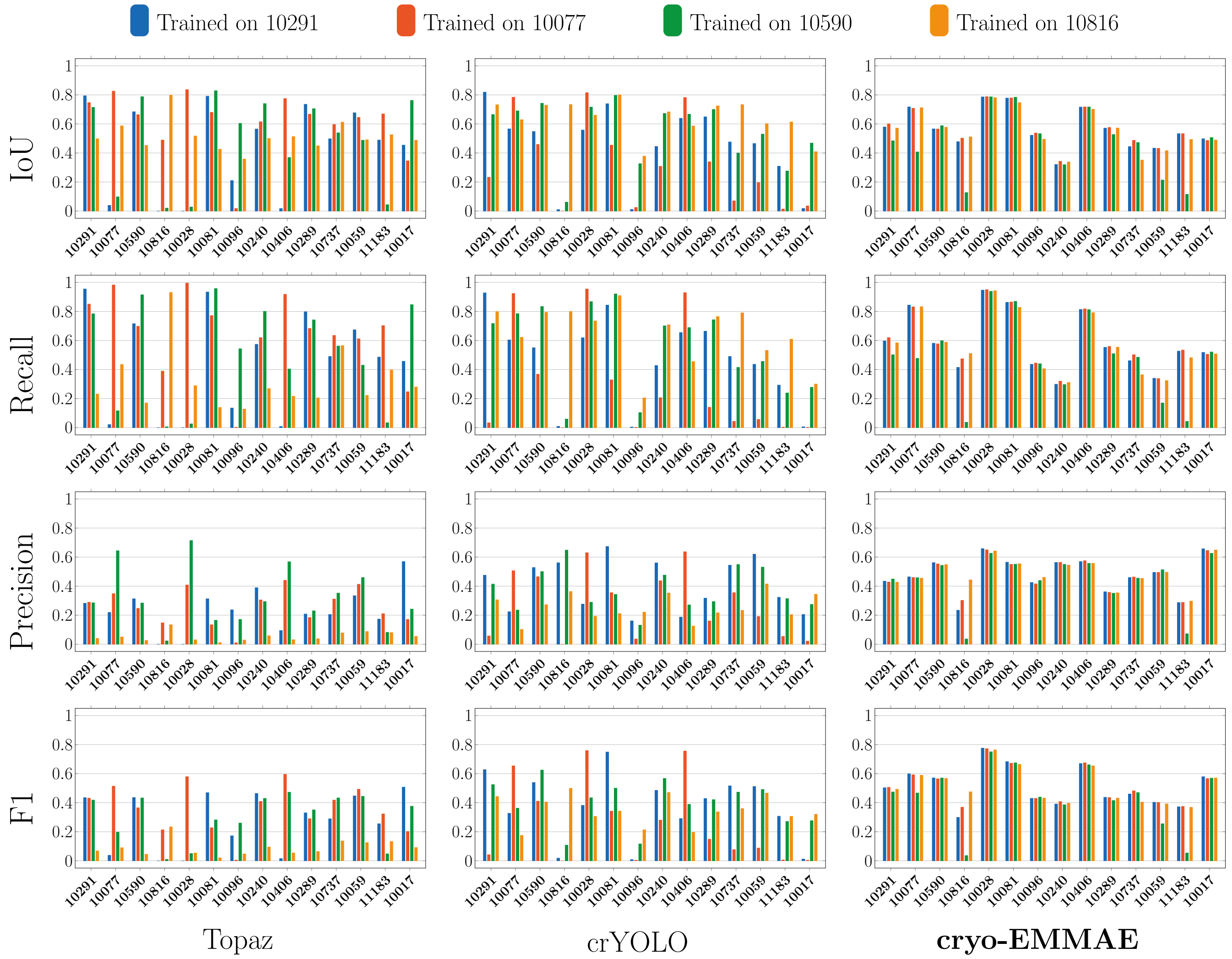

Figure S2: **Performance bar plots. Related to Table 1.** Bar plots depict the comparative performance of Topaz, crYOLO, and cryo-EMMAE across four key metrics (IoU, Recall, Precision, F1), trained on distinct datasets (10291, 10077, 10590, 10816), distinguished by color. Evaluation is conducted on 14 datasets, illustrated on the horizontal axis.

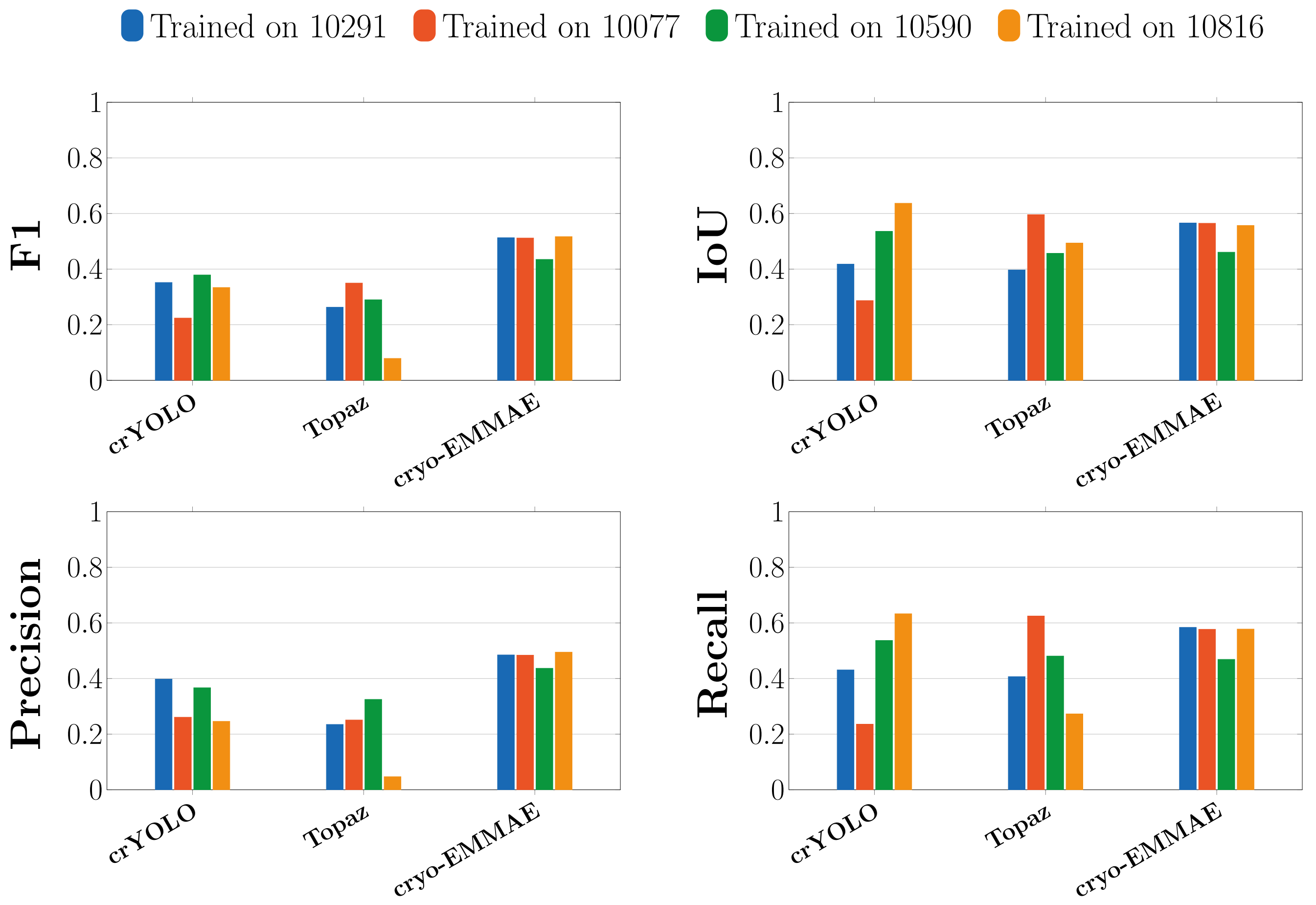

Figure S3: **Overall performance bar plots. Related to Table 1.** Bar plots show the comparative performance of Topaz, crYOLO, and cryo-EMMAE across four key metrics (IoU, Recall, Precision, F1), with each method trained on different datasets (10291, 10077, 10590, 10816), represented by distinct colors. The metrics are averaged over the 13 test datasets.

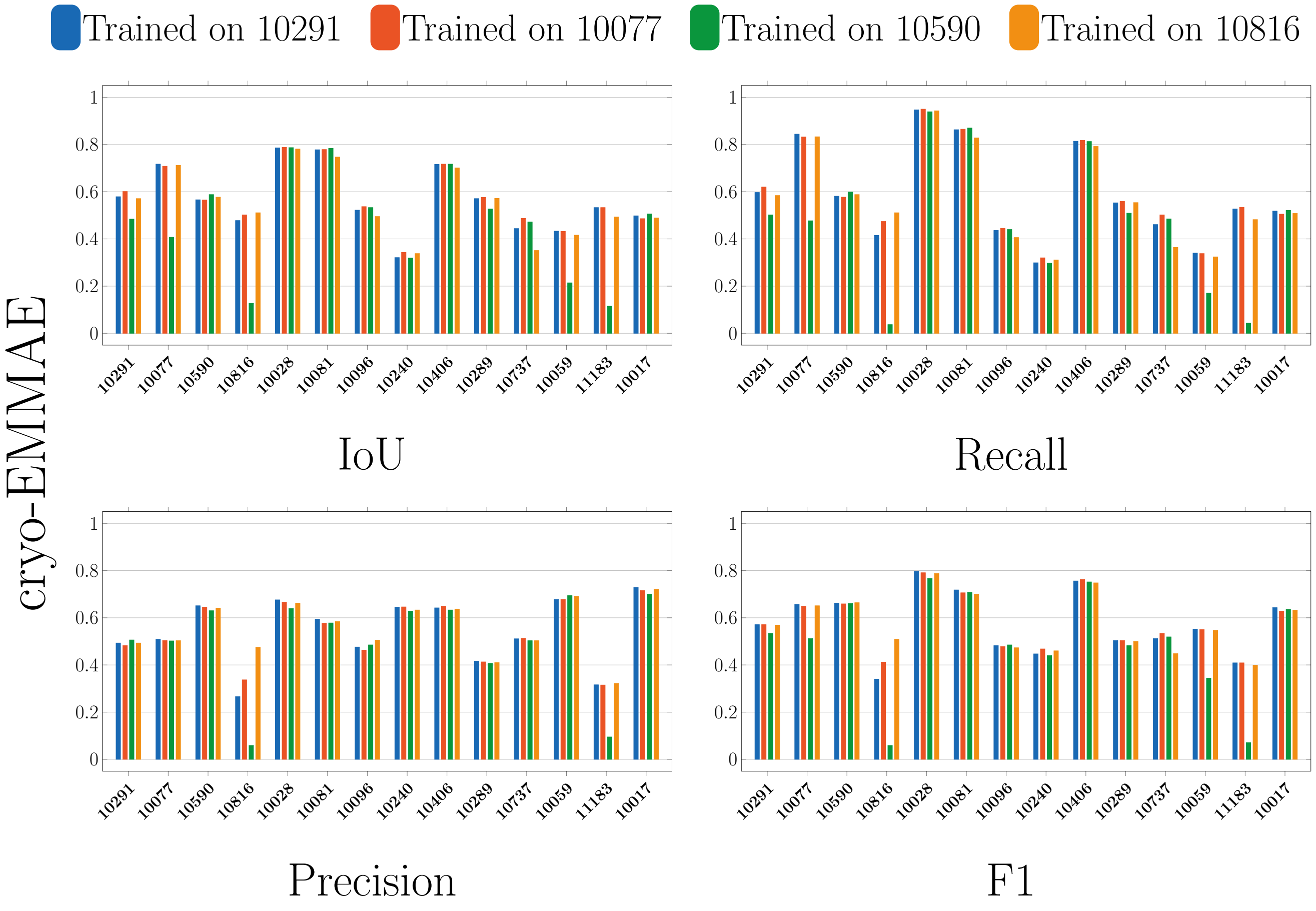

Figure S4: **Performance bar plots for 0.5 IoU threshold. Related to Table 1.** Performance of Cryo-EMMAE for IoU threshold of 0.5 across four metrics (IoU, Recall, Precision, F1), trained on distinct datasets (10291, 10077, 10590, 10816). Evaluation conducted on 14 datasets.

Table S3: **Mean distances of latent representations for particle and background regions. Related to Figure 3.** Mean distances are computed within Micrograph A, within Micrograph B, and across Micrographs A and B. Each block reports distances between region types (Particles or Background).

Micrograph	Region	Particles	Background
A (within)	Particles	3.08	3.32
	Background	3.32	3.16
B (within)	Particles	3.35	3.45
	Background	3.45	3.33
A $\rightarrow$ B (cross)	Particles	3.63	3.53
	Background	3.94	3.65

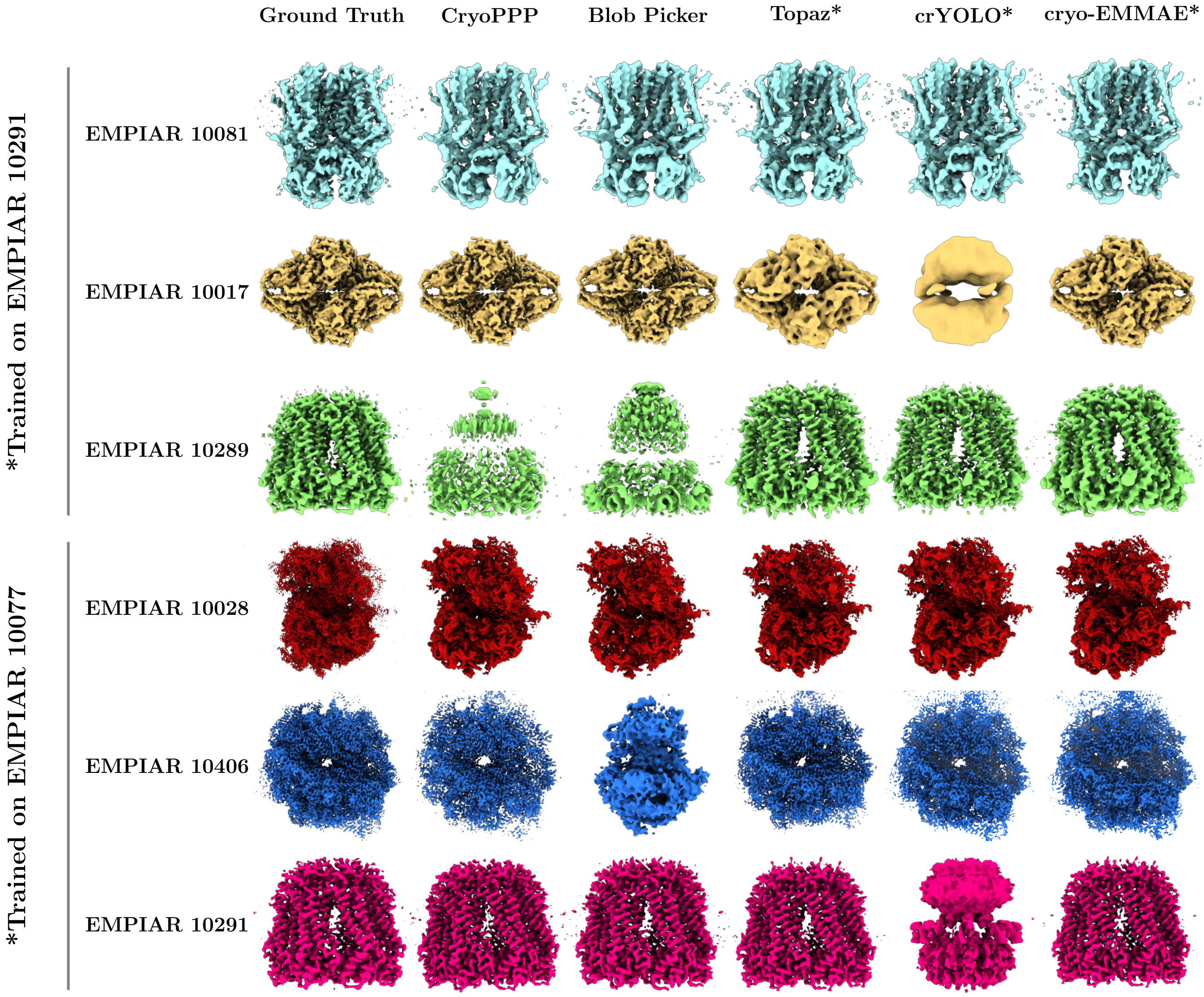

Figure S5: **Visualization of reconstructed density maps of ground truths and the four methods. Related to Table 2.** Each EMPIAR dataset is represented by a different color for clarity. For all columns except Ground Truth, reconstructed volumes were generated from a subset of approximately 300 micrographs, except for EMPIAR 10017, where only 84 micrographs were used. For the learning-based methods (Topaz, crYOLO, and cryo-EMMAE), the first three datasets were reconstructed using models trained on EMPIAR 10291, while the last three used models trained on EMPIAR 10077. The volumes reconstructed by the four methods (Blob Picker, Topaz, crYOLO, and cryo-EMMAE) include the 2D classification step, whereas the reconstructions of CryoPPP (annotation) particles do not include 2D classification.

Table S4: **Number of picked particles per reconstruction. Related to Table 2.** Number of picked perticles for eight different test datasets across four methods: Blob Picker, Topaz, crYOLO, and cryo-EMMAE. The last three methods were trained on datasets 10291 and 10077. The number of picked particles is reported both with and without 2D classification, with the percentage of retained particles included for the latter. Entries that did not successfully reconstruct the expected density map are highlighted in gray. Averaging is computed only for the correctly reconstructed entries.

Test Set	Trained on 10291								
	Without 2D Classification				With 2D Classification				
	Blob Picker	Topaz	crYOLO	cryo-EMMAE	Blob Picker	Topaz	crYOLO	cryo-EMMAE	CryoPPP GT
10028	32,102	0	19,272	35,997	29,067 (90.6%)	0 (-%)	17,147 (89.0%)	32,293 (89.7%)	25,050
10081	80,829	49,723	77,459	57,805	38,325 (47.4%)	38,019 (76.5%)	40,725 (52.6%)	36,828 (63.7%)	39,315
10017	50,160	4,057	268	37,552	45,445 (90.6%)	3,329 (82.1%)	232 (86.6%)	31,926 (85.0%)	49,178
11183	152,700	43,964	27,747	146,172	68,100 (44.6%)	32,624 (74.2%)	11,010 (39.7%)	54,218 (37.1%)	79,736
10289	93,384	119,992	85,657	91,127	48,178 (51.6%)	55,396 (46.2%)	41,608 (48.6%)	34,292 (37.6%)	61,455
10406	33,763	0	32,447	32,654	28,782 (85.3%)	0 (-%)	24,968 (86.1%)	27,922 (85.5%)	24,669
10077	70,990	0	28,929	56,808	22,546 (31.8%)	0 (-%)	19,597 (67.7%)	32,889 (57.9%)	31,834
Mean	73,418	31,105	38,826	65,445	40,063 (63.1%)	18,481 (69.7%)	22,184 (67.2%)	35,767 (65.2%)	44,462

Test Set	Trained on 10077								
	Without 2D Classification				With 2D Classification				
	Blob Picker	Topaz	crYOLO	cryo-EMMAE	Blob Picker	Topaz	crYOLO	cryo-EMMAE	CryoPPP GT
10028	32,102	33,016	32,022	36,589	29,067 (90.6%)	29,127 (88.2%)	29,127 (91.0%)	31,844 (87.0%)	25,050
10081	80,829	39,852	7,945	58,933	38,325 (47.4%)	15,237 (38.2%)	4,165 (52.4%)	38,674 (65.6%)	39,315
10017	50,160	518	426	37,742	45,445 (90.6%)	283 (54.6%)	175 (41.1%)	34,696 (91.9%)	49,178
11183	152,700	102,490	1,123	144,618	68,100 (44.6%)	60,733 (59.3%)	1,123 (100.0%)	54,077 (37.4%)	79,736
10289	93,384	92,627	18,597	92,627	48,178 (51.6%)	33,837 (36.5%)	10,267 (55.2%)	31,167 (33.7%)	61,455
10406	33,763	30,916	29,746	32,775	28,782 (85.3%)	29,793 (96.4%)	28,854 (97.0%)	28,804 (87.9%)	24,669
10291	172,240	73,632	20,153	135,795	105,731 (61.4%)	50,063 (68.0%)	14,253 (70.7%)	81,099 (59.7%)	99,765
Mean	87,883	53,293	15,716	77,011	51,947 (67.3%)	31,296 (63.0%)	12,566 (72.5%)	42,909 (66.2%)	54,167

Table S5: **Architecture parameters of ViT encoder/decoder and configuration parameters for cryo-EMMAE. Related to Figure 1.**

**Configuration**	**ViT Encoder**	**ViT Decoder**
Embedding Dimensions	192	128
Transformer Layers	14	7
Attention Heads	1	8
MLP Ratio	2.0	2.0
**General Configuration**		
Initial Image Shape	1024×1024	
Image's Number of Patches	256	
ViT Input Image Shape	64×64	
ViT Number of Patches	16	
ViT Patch Shape	4×4	
Optimizer	AdamW	
Base Learning Rate	1.0×10^{-3}	
Weight Decay	1.0×10^{-5}	
Optimizer Momentum	$\beta_1, \beta_2 = 0.9, 0.999$	
Batch Size	128	
Training Images	$\approx$ 46,000	
Mask Ratio	0.5	

Table S6: **Comparison of 3D reconstruction resolutions and the number of particles used between the EMPIAR published datasets and machine learning-based particle pickers. Related to Single Particle 3D Reconstructions subsection of the main manuscript.** Results are reported after one round of 2D classification and selection, with mean values also provided. The only exception is EMPIAR 10049 for Topaz, where two rounds of 2D classification were required.

Symmetry	EMPIAR	3D Reconstruction Resolution				Number of Particles			
		EMPIAR	Topaz	crYOLO	cryo-EMMAE	EMPIAR	Topaz	crYOLO	cryo-EMMAE
C1	10028	**3.20Å**	3.76Å	3.79Å	3.79Å ↑0.59Å	105,247	138,735	125,947	134,330
C2	10049	3.40Å	3.25Å	3.25Å	**3.21Å** ↓0.04Å	108,544	168,389	180,900	138,153
C3	10433	**3.22Å**	3.43Å	3.54Å	3.45Å ↑0.23Å	54,395	59,872	53,011	55,733
D2	10955	**2.16Å**	2.50Å	3.04Å	2.49Å ↑0.33Å	88,731	91,049	9,903	40,418
	Mean	**3.00Å**	3.24Å	3.40Å	3.24Å ↑0.24Å	89,229	114,511	92,440	92.158

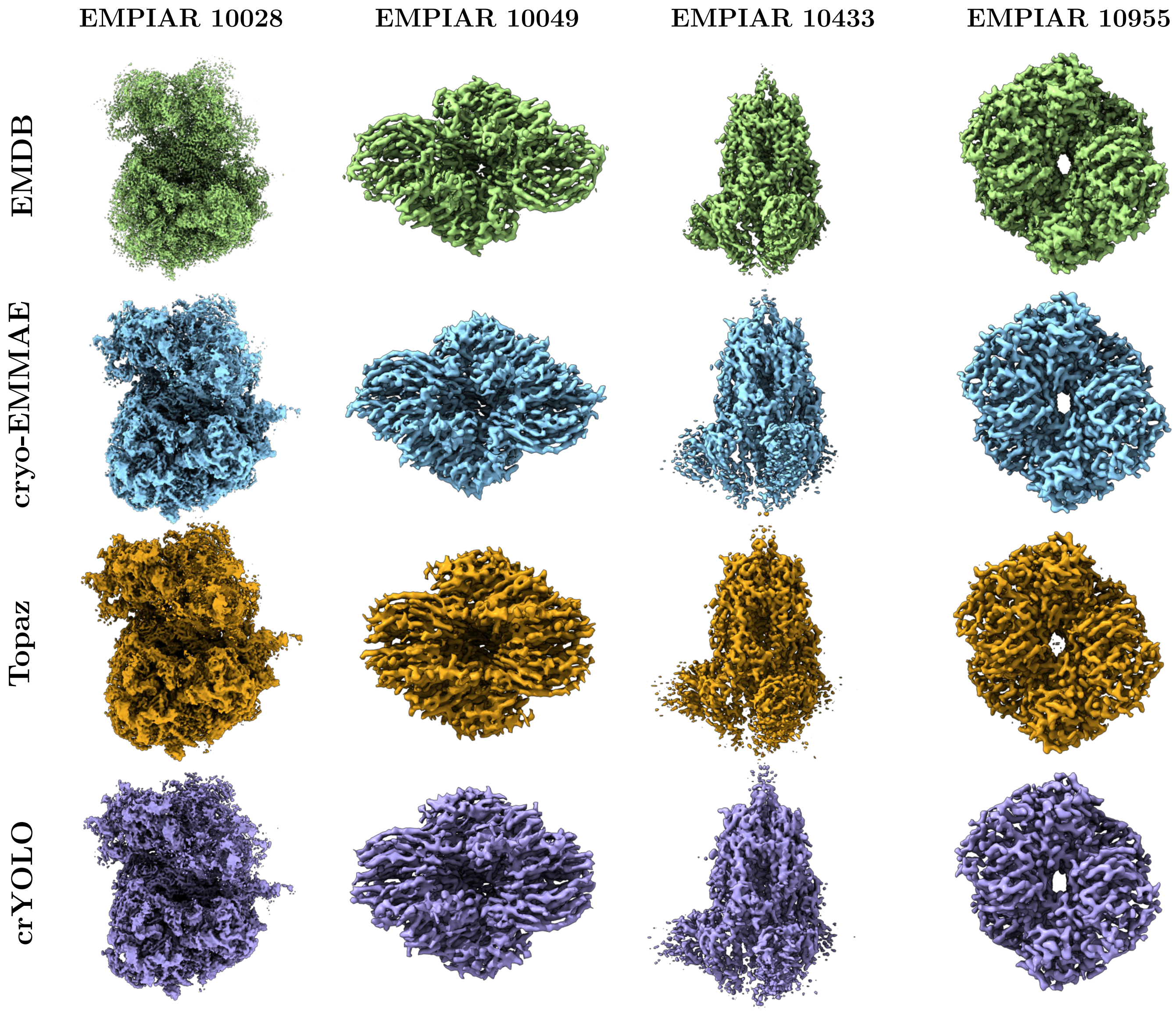

Figure S6: **Visualization of 3D reconstructed density maps from the published volumes in EMDB, cryo-EMMAE, Topaz, and crYOLO evaluating across four different EMPIAR datasets. Related to Single Particle 3D Reconstructions subsection of the main manuscript.** The three machine learning methods have been **trained on 20 EMPIAR datasets**. EMDB maps are shown in green, those generated using cryo-EMMAE in blue, Topaz in gold, and crYOLO in purple. Maps produced by the machine learning methods underwent only one round of 2D classification and were not subjected to any post-processing of the 3D reconstructed map. The only exception is EMPIAR 10049 for Topaz, where two rounds of 2D classification were required. The density threshold is selected using the ChimeraX sdlevel command, which determines the threshold based on standard deviations (SD) from the mean for each 3D density map.

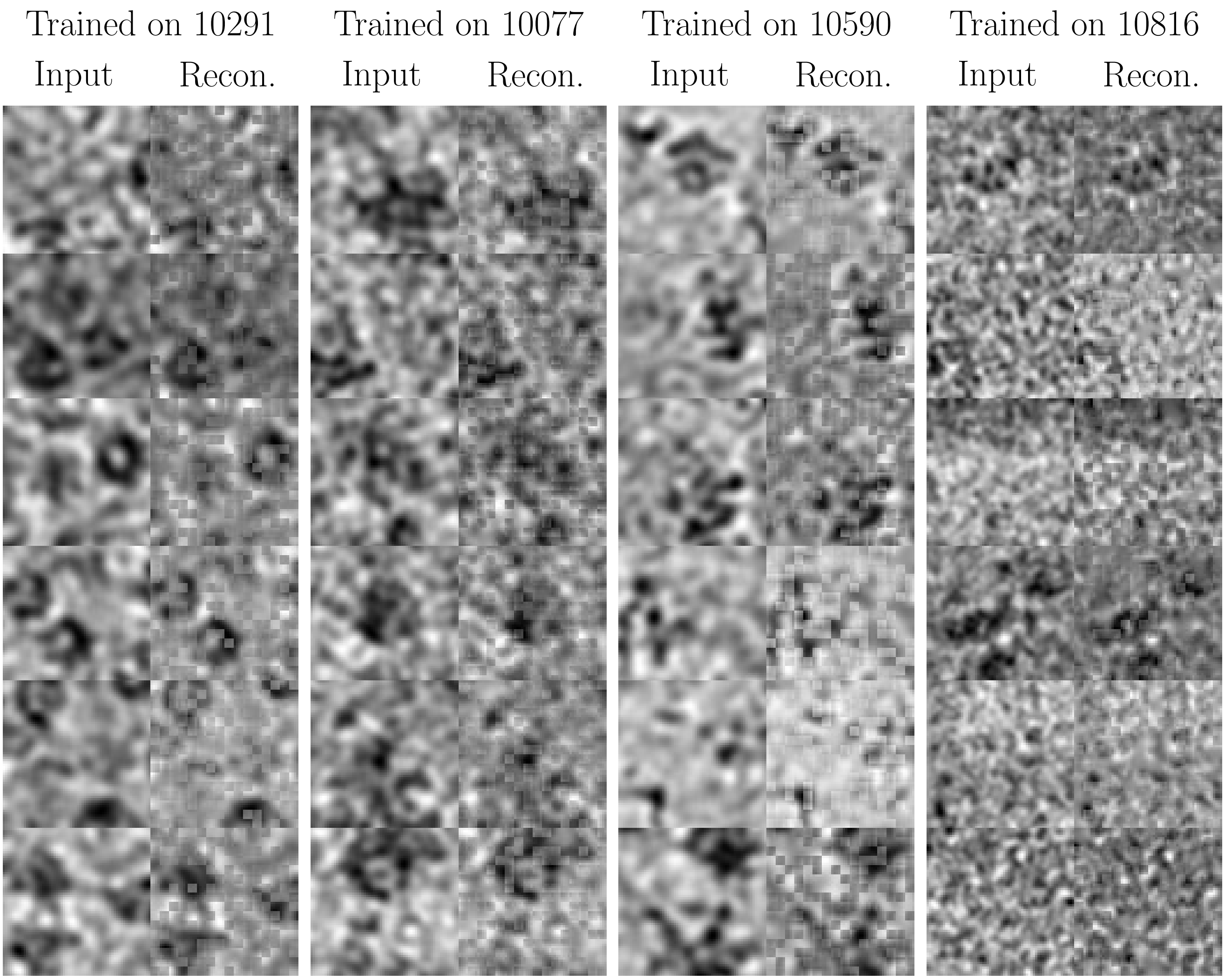

Figure S7: **Examples of input and reconstructed images from Cryo-EMMAE with a mask ratio of 0.5. Related to Figure 1.** Models trained independently on four different EMPIAR datasets: 10291, 10077, 10590, and 10816.
