# Supplementary material for: Self-supervised learning for generalizable particle picking in cryo-EM micrographs
Source: Cell Rep Methods. 2025 Jul 7;5(7):101089. doi: 10.1016/j.crmeth.2025.101089 (PMC12296464; doi:10.1016/j.crmeth.2025.101089)
Supplement: Document S2. Article plus supplemental information [file mmc2.pdf]

Cell Reports
Methods

Article

Self-supervised learning for generalizable particle picking in cryo-EM micrographs

Graphical abstract

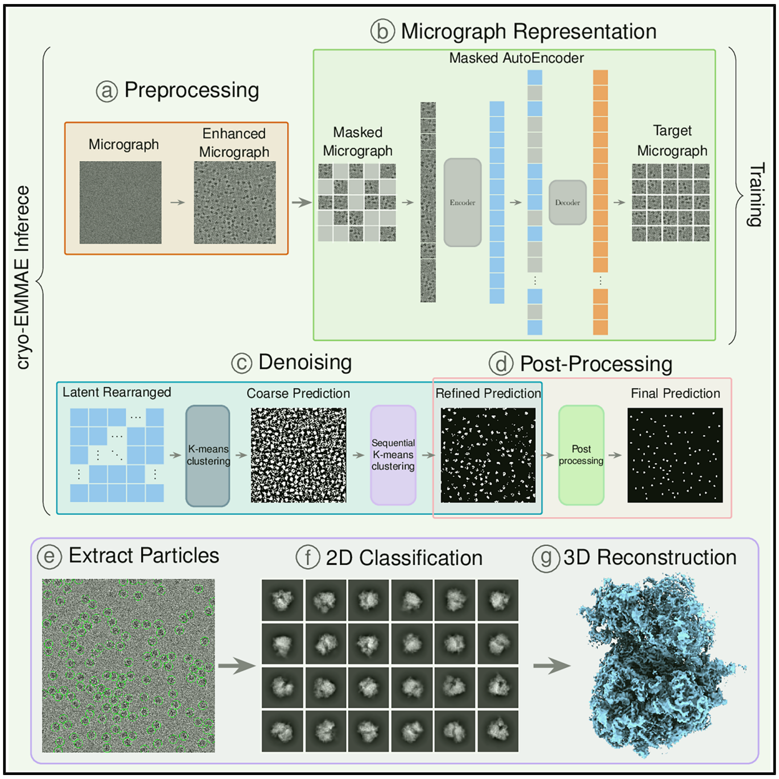

Highlights

- We introduce cryo-EMMAE, a self-supervised method for particle picking
- Our method generalizes well to real-world experiments not seen during training
- We validate our method on challenging micrographs containing cell extract samples

Authors

Andreas Zamanos, Panagiotis Koromilas, Giorgos Bouritsas, Panagiotis L. Kastritis, Yannis Panagakis

Correspondence

a.zamanos@di.uoa.gr

In brief

Zamanos et al. introduce a self-supervised particle picking method for cryoelectron microscopy (cryo-EM) that overcomes the need for annotations. Leveraging masked autoencoders to learn micrograph features, the pipeline clusters pixels into particle or background classes, allowing for robust and accurate particle picking across diverse cryo-EM experiments.

Zamanos et al., 2025, Cell Reports Methods *5*, 101089
July 21, 2025 © 2025 The Authors. Published by Elsevier Inc.
https://doi.org/10.1016/j.crmeth.2025.101089

CellPress

Cell Reports Methods

CellPress
OPEN ACCESS

Article

Self-supervised learning for generalizable particle picking in cryo-EM micrographs

Andreas Zamanos,[1,2,7,*] Panagiotis Koromilas,[1] Giorgos Bouritsas,[1,2] Panagiotis L. Kastritis,[3,4,5,6] and Yannis Panagakis[1,2]

[1]Department of Informatics and Telecommunications, National and Kapodistrian University of Athens, 16122 Athens, Greece
[2]Archimedes Unit, Athena Research Center, 15125 Athens, Greece
[3]Department of Integrative Structural Biochemistry, Institute of Biochemistry and Biotechnology, Martin Luther University Halle-Wittenberg, Kurt-Mothes-Straße 3, 06120 Halle/Saale, Germany
[4]Biozentrum, Martin Luther University Halle-Wittenberg, Weinbergweg 22, 06120 Halle/Saale, Halle, Germany
[5]Institute of Chemical Biology, National Hellenic Research Foundation, 11635 Athens, Greece
[6]Interdisciplinary Research Center HALOmem, Charles Tanford Protein Center, Martin Luther University Halle-Wittenberg, Kurt-Mothes-Straße 3a, 06120 Halle/Saale, Germany
[7]Lead contact
*Correspondence: a.zamanos@di.uoa.gr
https://doi.org/10.1016/j.crmeth.2025.101089

MOTIVATION Cryoelectron microscopy (cryo-EM) has become a vital technique in structural biology, enabling the determination of protein structures at high resolution. A critical step in this process is "particle picking," which involves the localization of protein particles in cryo-EM micrographs. The accuracy of particle picking strongly influences the quality of the 3D protein structure, since the identified particle projections are used to reconstruct the 3D electron density map. To date, all automated machine learning-based methodologies for this crucial task are based on techniques that rely on human supervision thus leading to three main limitations, namely that systems (1) require costly annotated datasets, (2) cannot generalize to unseen data distributions such as different proteins or cryo images captured under different laboratory settings, and (3) demand fine-tuning and further supervision to adapt to unseen data. To overcome these challenges, we propose cryo-EMMAE, the first self-supervised particle picker that entirely eliminates the need for annotations while demonstrating strong generalization capabilities, even in the context of highly heterogeneous specimens, such as native cell extracts.

SUMMARY

We present cryoelectron microscopy masked autoencoder (cryo-EMMAE), a self-supervised method designed to overcome the need for manually annotated cryo-EM data. cryo-EMMAE leverages the representation space of a masked autoencoder to pick particle pixels through clustering of the MAE latent representation. Evaluation across different EMPIAR datasets demonstrates that cryo-EMMAE outperforms state-of-the-art supervised methods in terms of generalization capabilities. Importantly, our method showcases consistent performance, independent of the dataset used for training. Additionally, cryo-EMMAE is data efficient, as we experimentally observe that it converges with as few as five micrographs. Further, 3D reconstruction results indicate that our method has superior performance in reconstructing the volumes in both single-particle datasets and multi-particle micrographs derived from cell extracts. Our results underscore the potential of self-supervised learning in advancing cryo-EM image analysis, offering an alternative for more efficient and cost-effective structural biology research. Code is available at https://github.com/azamanos/Cryo-EMMAE.

INTRODUCTION

Cryoelectron microscopy (cryo-EM) has transformed structural biology by facilitating the imaging of biological macromolecules at near-atomic resolution. In a standard cryo-EM experimental protocol, a purified protein sample is rapidly frozen in a thin layer of vitreous ice, to preserve their native structures and minimize radiation damage. The frozen sample is then imaged in an

© 2025 The Authors. Published by Elsevier Inc.
This is an open access article under the CC BY license (http://creativecommons.org/licenses/by/4.0/).

electron microscope, producing two-dimensional (2D) projections of the protein randomly distributed in images called micrographs. Each micrograph contains numerous randomly oriented copies of the molecule of interest, the so-called particles.[1–3] Despite its importance, the analysis of cryo-EM data presents several unique challenges that arise from the nature of the imaging technique and the biological samples being studied.[4,5]

One of the most critical steps in cryo-EM data processing is particle picking,[6] the process of selecting individual particles from noisy and heterogeneous micrographs. This step is challenging due to several inherent factors in cryo-EM data. First, Cryo-EM micrographs typically exhibit a low signal/noise ratio due to the low electron dose that is used during imaging to minimize radiation damage on the delicate biological samples.[7] Consequently, the high noise levels make the particles almost indistinguishable from the background.[8] Second, the appearance of particles in cryo-EM micrographs is highly variable. This variability is a result of differences such as particle orientation, conformational states, micrographs with multi-proteins samples[9] and the presence of artifacts such as ice contamination. The heterogeneity of particle appearance further complicates the particle-picking, as it becomes more challenging to establish consistent criteria for identifying and selecting particles. Third, cryo-EM datasets often have different parameters during data collection, such as the accelerating voltage, the total electron exposure dose, and vitreous ice thickness. These variations can lead to deviations in the appearance and contrast of particles across different datasets. Additionally, manual annotation of particles is a time-consuming, laborious, and prone to human bias and inconsistencies process.

The main objective of cryo-EM analysis is to produce the highest possible resolution for the protein's 3D density map from a given dataset of micrographs. A high-resolution 3D map provides more detailed atomic positions of the protein, increases the certainty of the atomic structure, and thus enhances its credibility. Various computational approaches have been proposed to automate particle picking from developing traditional methods that are either template based[10,11] or template free,[12–16] to supervised deep learning techniques that are based on one of semantic segmentation,[17–21] classification,[22–27] or object detection.[28–30] However, these methods (1) still require a substantial amount of manually picked particles for training and fine-tuning, thus creating a fundamental bottleneck in the cryo-EM workflow. Along with this demand for costly expert annotated data, we empirically observe that state-of-the-art approaches based on classification or object detection (2) struggle to generalize to unseen data and experimental conditions. Additionally, all existing deep learning-based methods are designed to (3) work on micrographs containing purified samples of a single protein. This constraint prevents their application to more challenging and promising scenarios involving multi-protein micrographs, which could reveal complex interactions inherent to intracellular processes.[9,31,32] These three core challenges render existing methods unsuitable for real-world applications, especially in laboratory settings where data availability is limited. In such cases, practitioners are unable to effectively use pre-trained networks or train models from scratch.

In this work, we make a first step toward alleviating the reliance on annotations and provide a potential alternative to this limited resource setup. We introduce cryoelectron microscopy masked autoencoder (cryo-EMMAE), the first self-supervised particle picking method. This approach, as illustrated in Figure 1, leverages a masked autoencoder (MAE) to segment micrographs by clustering the MAE latent representation space. Our method's self-supervised nature arises from the learning process of MAE, which reconstructs masked patches of input micrographs using only the original images as both input and target data. Through this process of image reconstruction, without requiring any labels or annotations, the model learns useful features and patterns, distilling this information into a latent representation. At inference time, these distilled representations, learned purely from unlabeled micrographs, are utilized for micrograph segmentation. Initially, a clustering algorithm trained on the training data is used to differentiate the background from the particle latent space shared across all micrographs. Subsequently, hierarchical clustering is applied to each micrograph to progressively filter micrograph-specific noise from particles.

We trained cryo-EMMAE along two state-of-the-art deep learning methods, Topaz[23] and crYOLO,[28] from scratch using four annotated EMPIAR datasets provided by CryoPPP.[33] The models were then evaluated on these datasets as well as 10 additional EMPIAR datasets, which were not seen during training. These evaluation datasets were chosen to represent a wide variety of protein types, functions, subcellular locations, organism origins, shapes, sizes, noise characteristics, and protein concentrations in the micrographs.

Results show that, unlike existing methods, cryo-EMMAE exhibits excellent generalization, delivering stable performance across all evaluation sets, regardless of the pretraining data.

Additionally, we report that, for a given dataset, cryo-EMMAE converges toward its optimal performance with just 5 micrographs (equivalent to 1,280 training images for the MAE), indicating that using more data from the same data source does not significantly improve performance. This makes our method annotation-free, independent of the pretraining data types, and trainable with a minimal number of micrographs. We further demonstrate that protein reconstructions generated using particles picked by cryo-EMMAE outperform those produced by cryoSPARC's Blob Picker,[15] Topaz, and crYOLO, even for proteins not encountered during training. Finally, we present promising results on experiments based on cell extracts, when methods are trained with extended training dataset.

The main contributions of this paper are summarized as follows.

(1) We introduce cryo-EMMAE the first self-supervised method for particle picking in cryo-EM data that does not require any form of annotation.
(2) Cryo-EMMAE demonstrates stable generalization capabilities when applied to unseen data distributions and outperforms supervised methods, highlighting the effectiveness of our unsupervised approach in handling diverse cryo-EM datasets.
(3) Our method achieves exceptional generalization even when trained on a limited number of micrographs. This is especially beneficial in situations where annotated data are limited or difficult to acquire.

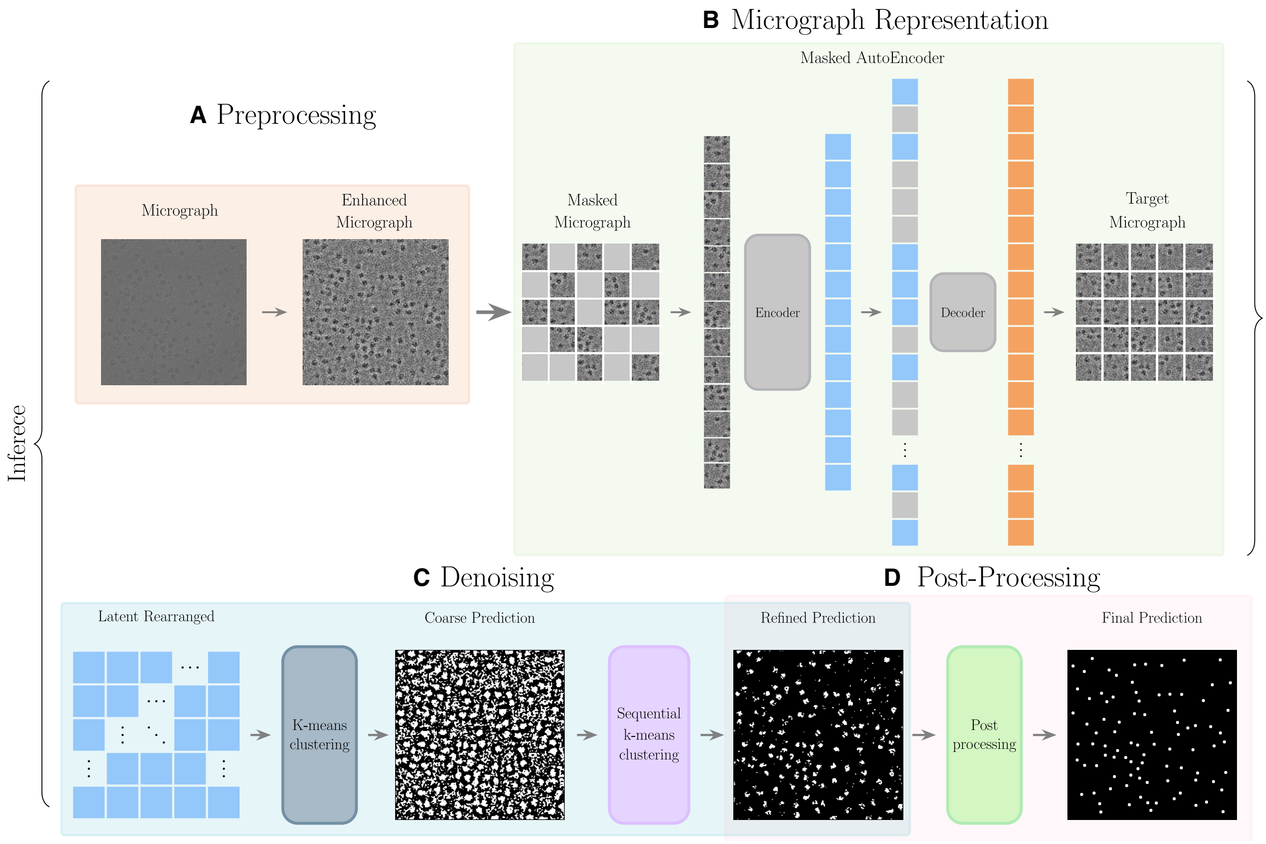

Figure 1. The cryo-EMMAE pipeline

The cryo-EMMAE pipeline starts with an input micrograph and follows these steps:

(A) Pre-processing: the micrograph undergoes normalization of background noise to minimize correlation with experimental parameters and is filtered to enhance particle contrast.

(B) Micrograph representation: patches are extracted from the pre-processed micrograph and used to map it onto the MAE representation space.

(C) Denoising: the resulting embeddings form a smaller image where a k-means trained on the train set identifies pixels with the lowest noise levels. These images undergo further denoising through micrograph-specific hierarchical clustering.

(D) Post-processing: convolution-based smoothing is applied on the predictions of the particle centers with greater accuracy.

(4) We are the first to apply our method to micrographs with samples from cell extracts, i.e., multi-particle samples that have not undergone any over-expression and purification. In this challenging setup, we demonstrate the superior performance of cryo-EMMAE.

(5) We show the effectiveness of segmenting through clustering latent representations learned from cryo-EM data. By incorporating the representation clustering, cryo-EM-MAE can effectively distinguish the underlying protein structure and patterns in the presence of noise.

RESULTS

In this section, we compare three commonly used methods for particle picking: (1) Blob Picker,[15] a traditional template-free approach that picks particles by searching for Gaussian signals and does not rely on annotated data but requires active human supervision in hyper-parameter searching, (2) Topaz,[23] a state-of-the-art classification-based method, and (3) crYOLO,[28] the highest-performing object detection-based method across various particle picking tasks and setups. For evaluation, we use subsets of the CryoPPP dataset for training and testing, as described in Dhakal et al.[33] Experimental details are listed in experimental setup. Our experimental results involve training on four different datasets separately and evaluating the performance of each model on 14 datasets, as detailed in Tables 1 and S1 and illustrated in Figure S2. Additionally, we report results on a particularly challenging dataset (EMPIAR: 10892), which contains data from cell extracts,[9] with the machine learning methods trained on 20 EMPIAR datasets in total. Finally, we apply cryo-EMMAE to real-world scenarios using the complete set of micrographs from 6 EMPIAR experiments and compare the resulting 3D reconstructions against published maps.

Comparison under the supervised setup

An initial point of interest is the supervised scheme, where each method is evaluated on the dataset (one of EMPIAR: 10291, 10077, 10590, 10816) that it has been trained on. First, results from Table 1 indicate that our method demonstrates superior performance over Topaz with respect to the F1 metric. Additionally, it

Table 1. Each method is trained on four different datasets, and their generalization performance is evaluated on 14 EMPIAR experiments

Trained on	Method	IoU	Recall	Precision	F1
10291	Topaz	0.425	0.446	0.238	0.276
	CrYOLO	0.447	0.467	0.404	0.372
	cryo-EMMAE	**0.567**	**0.585**	**0.481**	**0.512**
10077	Topaz	**0.612**	**0.651**	0.258	0.362
	CrYOLO	0.322	0.285	0.279	0.255
	cryo-EMMAE	0.575	0.596	**0.482**	**0.518**
10590	Topaz	0.481	0.512	0.322	0.300
	CrYOLO	**0.551**	**0.558**	0.376	0.397
	cryo-EMMAE	0.470	0.479	**0.444**	**0.444**
10816	Topaz	0.515	0.320	0.053	0.090
	CrYOLO	**0.644**	**0.645**	0.254	0.346
	cryo-EMMAE	0.554	0.573	**0.492**	**0.514**

The table reports the mean values of four evaluation metrics across the test set: (1) Intersection over Union (IoU), (2) recall (a prediction is a true positive if IoU $\geq$ 0.6), (3) precision, and (4) F1 score. For complete per-experiment results, refer to Table S1. Bold values indicate the best performance for each metric.

closely matches crYOLO in three out of the four cases, with the exception being dataset EMPIAR: 10291. However, both supervised methods outperform cryo-EMMAE in the Intersection over Union (IoU) metric. Therefore, in the supervised setting, while our method is comparable in identifying particles with good precision, it exhibits inferior performance in predicting particles and their centers (IoU) compared with the supervised methods.

Generalization ability

The results reported in Table S1 and Figure S2 suggest that supervised methods struggle to generalize to unseen data distributions. Across all metrics reported, their performance frequently drops to exceptionally low levels. Notably, both Topaz and crYOLO often exhibit near-zero values for F1 score, IoU, precision, or recall metrics. Even for cases of non-zero performance, they are still inferior to the ones obtained with supervised training. In contrast, cryo-EMMAE demonstrates notable cross-dataset generalization capabilities. Its performance consistently remains nearly stable across various evaluation datasets and models trained on different datasets.

This suggests that our method effectively mitigates the impact of dataset-specific noise levels and characteristics in micrographs. These findings imply that cryo-EMMAE can learn the necessary invariances irrespective of the experimental nuances inherent in cryo-EM procedures. As shown in Table 1, cryo-EMMAE's mean F1 and precision scores across all four training paradigms are superior to both Topaz and crYOLO. However, while the mean IoU and recall values are comparable across all three methods, cryo-EMMAE lags behind in three out of four training setups.

Performance scaling vs. training set size

In Figure 2A, we highlight an interesting characteristic of cryo-EMMAE: our approach achieves strong performance after training on just 5 micrographs. Each micrograph is divided into 256 images (since they are resized to a 1,024 $\times$ 1,024 shape), meaning that, when trained on 5 micrographs, our method is effectively trained on 1,280 images in total. We hypothesize that this behavior results from the randomness inherent in the masking process during our preprocessing pipeline, which helps mitigate the influence of experimental factors, such as ice thickness, on micrograph noise. This noise normalization makes the data less variable at a local scale, while reconstructing random patches can amplify this variability.

Role of micrograph-specific clustering

In Figure 2B, we illustrate the impact of the number of clusters that are used in the micrograph-specific clustering process by reporting aggregated results (IoU, recall, precision, and F1 averaged across the 14 datasets) for cryo-EMMAE trained on the dataset EMPIAR: 10029. Our ablation study reveals that different number of clusters directly affects the final performance. These findings highlight two observations: (1) as the number of clusters increases, precision improves while recall decreases, indicating that the clustering process filters out more background pixels but also eliminates some particle positions and (2) the selection of five clusters for the micrograph-specific clustering process (described in detail in inference) optimizes the F1 score.

Latent space analysis

The latent representations of a micrograph are the feature vectors extracted from each micrograph patch by the MAE (see Figure 1B for the encoder output of the MAE). These latent representations, which are vectors of length 192, encode essential information about the input patches and are used to cluster and segment the micrograph into particles and background regions. Latent representations extracted from a specific micrograph tend to be more similar to each other than to those from different micrographs, as they originate from the same experimental conditions, including imaging parameters, noise characteristics, and particle distributions.

To visualize the discriminative capability of the latent space for micrograph pixels, we performed principal-component analysis on the latent representations of particle and background pixels. The first two principal components were plotted for six micrographs from different EMPIAR datasets. The results, shown in Figure 3, clearly demonstrate separation between background and particle pixels. To ensure visual balance, an equal number of data points were sampled from particles and background. These latent representations were obtained from the cryo-EMMAE model trained on the EMPIAR: 10291 dataset. In Table S3, we compute the Euclidean distances between latent representations of particles and background within the same micrographs and across two selected micrographs (A and B). The results show that particle regions within a micrograph have significantly lower distances between themselves than when compared with background regions, and vice versa. Additionally, intra-micrograph distances are consistently lower than inter-micrograph distances, indicating greater similarity within each micrograph. This supports the notion of micrograph-specific latent representations.

CellPress
OPEN ACCESS

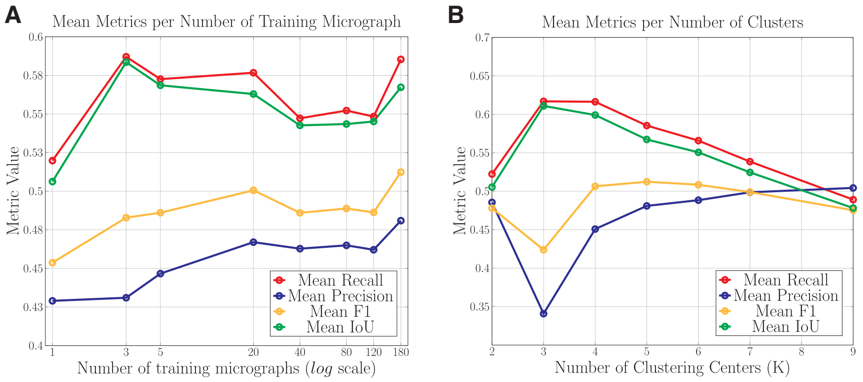

Figure 2. Performance metrics of the ablation study for cryo-EMMAE

The model was trained on the EMPIAR: *10291* dataset and evaluated across 14 datasets, with the mean values computed.

(A) The mean IoU, recall, precision, and F1 scores are plotted against the number of training micrographs.

(B) Presents the mean IoU, recall, precision, and F1 scores relative to the number of clusters (K) used during post-processing.

Single-particle 3D reconstructions

To further assess the particle-picking performance of each method, we performed 3D reconstructions on eight test datasets using models trained on two datasets (EMPIAR: 10291, 10077). The evaluation included reconstructions using blob picking, which resembles an unsupervised approach, and reconstructions based on CryoPPP annotation particles. All electron density maps were generated using CryoSPARC v.4.4.0.[15] Two workflows were used: the first involved *ab initio* 3D reconstruction followed by homogeneous refinement using the complete set of picked particles, while the second included 2D classification and selection of the best classes before reconstruction and refinement. For test datasets EMPIAR: 10081, 10017, 10289, 10291, the corresponding symmetries C4, D2, C8, and C8 were imposed during homogeneous refinement. For more details in 3D reconstruction procedures see 3D reconstruction methodology.

The best-resolution reconstruction for each method across eight different EMPIAR datasets is reported in Table 2, while the number of particles used to produce these reconstructions are presented in Table S4. Resolutions of 3D density maps that failed to reconstruct the volumes correctly are underlined, while the best resolution reconstructions for each test set and workflow (with and without 2D classification) are highlighted in bold. In Table 2 the average resolution across all reconstructions is calculated in the Mean row, by assigning a penalty value of 10 Å for test sets that were not correctly reconstructed. The Rec. Mean row reports the average resolution using only correctly reconstructed sets and indicates the percentage of successful reconstructions. Reconstructions using ground truth particles from CryoPPP do not involve 2D classification or class selection, as these particles are provided as a pre-filtered, optimal particle set, that is clean of any noise.

The results in Table 2 indicate that cryo-EMMAE not only picks particles that are used to successfully reconstruct most 3D density maps for both workflows but also reports the best mean resolution, significantly outperforming the second-best method, Blob Picker. On average, the differences in resolution between cryo-EMMAE and Topaz are approximately 2.9 and 1.2 Å for the EMPIAR: 10291, 10077 training schemes, respectively. These values were computed as the average resolution differences between the methods, both with and without 2D classification. Similarly, the corresponding differences with crYOLO are approximately 1.7 and 3.7 Å. An interesting result is that, when reconstructing using 2D classification, cryo-EMMAE reconstructions exhibit higher resolution compared with the ground truth picks provided by CryoPPP's annotation dataset, by reporting a difference of 1.71 and 1.05 Å for EMPIAR: 10291, 10077, respectively. Additionally, the CryoPPP particle set failed to reconstruct correctly two out of eight EMPIAR datasets. When these CryoPPP particle sets were 2D classified and further cleaned, the reconstructions were corrected, reaching resolutions of 3.65 Å for EMPIAR: 10289 and 5.29 Å for EMPIAR: 10077, with particles retention rates of 49% and 84%, respectively. This phenomenon probably occurs because CryoPPP annotations aim to include as many particles as possible, even those of lower resolution, prioritizing the training of machine learning algorithms over the reconstruction resolution of the particle set. In the discussion, we further elaborate on our opinion regarding annotations of particles.

Figure S4 presents 6 different 3D reconstructions across the 4 methods, along with ground truth volumes (produced using the entire EMPIAR datasets, rather than a subset of 300 micrographs) and CryoPPP reconstructions. The figure also highlights failed reconstructions for CryoPPP (EMPIAR: 10289), Blob Picker (EMPIAR: 10289), and crYOLO (EMPIAR: 10017, 10291). cryo-EMMAE on the other hand demonstrates consistent reconstruction performance across the 6 test datasets.

Finally, we performed 3D reconstructions on four EMPIAR datasets using Topaz, crYOLO, and cryo-EMMAE models trained on 20 EMPIAR datasets provided by CryoPPP, the results are presented in Figure S6 and Table S6. The reported resolutions in Table S6 indicate that Topaz and cryo-EMMAE perform similarly, while crYOLO ranks last, with a small difference of 0.26 Å. In Figure S6, we present the visualization of 3D reconstructions for the three methods compared with the published structures. The figure reveals minor differences between the reconstructions, except for EMPIAR: 10049, where cryo-EMMAE appears to reconstruct the density map more accurately than Topaz and crYOLO. Notably, for EMPIAR: 10049, Topaz required two rounds of classification to properly reconstruct the density map. Overall, we observed that Topaz selected substantially more particles than the other two methods, making the analysis significantly more time-consuming.

In conclusion, our experiments show that cryo-EMMAE, Topaz, and crYOLO achieve comparable performance when trained on the same extended training set. The key difference is that Topaz and crYOLO require annotated data, which can

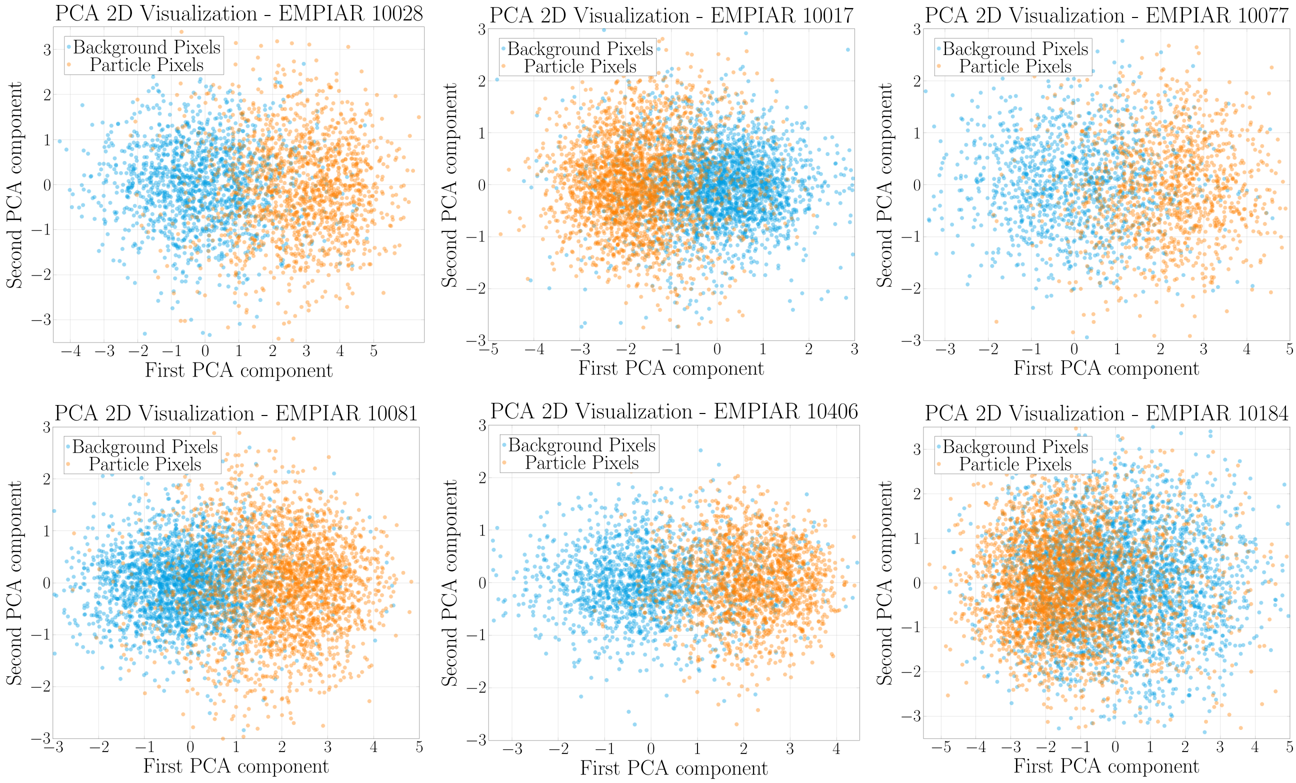

Figure 3. Latent space visualization through principal-component analysis for cryo-EMMAE's latent representations projected onto the first two principal components

The subplots correspond to different micrographs from six datasets (EMPIAR: 10028, 10017, 10077, 10081, 10406, 10184). Latent representation data points for background pixels are shown in cyan, while those for particle pixels are shown in orange.

be difficult to obtain in large quantities. In contrast, our main contribution is demonstrating that cryo-EMMAE can match the performance of these state-of-the-art supervised methods without relying on labeled data, making it a more practical and scalable solution.

Multi-particle 3D reconstructions

To further show the generalization ability of our method on unseen setups, we evaluated the four methods on a significantly challenging dataset that includes micrographs from cell extracts of *Chaetomium thermophilum* presented in Skalidis et al. [9] (EMPIAR: 10892). In this case, Topaz, crYOLO, and cryo-EMMAE were trained on an extensive dataset comprising 20 EMPIAR datasets from cryoPPP, in contrast to the single-particle 3D reconstruction experiments, where each method was trained separately on a single EMPIAR dataset. In this study, we reconstructed four different proteins: the pre-60S ribosomal subunit, fatty acid synthase (FAS), the E2 core of the oxoglutarate dehydrogenase complex (OGDHc), and the E2 core of the pyruvate dehydrogenase complex (PDHc). Using 2,808 micrographs, the study achieved resolutions of 4.52, 4.47, 4.38, and 3.84 Å for the pre-60S, FAS, OGDHc, and PDHc, respectively.

For our evaluation, a subset of 854 micrographs was selected from the original dataset. These were chosen as the top 300 micrographs containing most of the particles for each protein. Some micrographs overlapped across proteins, leading to a total of 854 instead of 1,200 micrographs (4 $\times$ 300). The three machine learning methods were trained on a large training dataset of 1,950 micrographs from 20 different EMPIAR datasets. Data preprocessing steps, the number of training epochs, and checkpoint selection follow the methodology detailed in experimental setup.

The resolution results of the 3D reconstructions are presented in Table 3, while visualizations of the 3D electron density volumes are illustrated in Figure 4. For the reconstructions, we performed two rounds of classification and selection with 300 classes each to better isolate the protein classes. The total number of particles selected by each method was 747,225 for blob picking, 249,473 for Topaz, 92,595 for crYOLO, and 253,045 for cryo-EMMAE. The two classification rounds required approximately 25.5, 10.3, 4.5, and 10.5 GPU hours, respectively, using CryoSPARC v.4.4.0.[15]

As shown in Table 3, cryo-EMMAE not only, unlike other methods, successfully reconstructs all protein densities but also achieves the best mean resolution of 5.89 Å, outperforming the second-best, Topaz (6.91 Å). The average resolution per method incorporates a penalty of 10 Å for each unreconstructed entry. Furthermore, cryo-EMMAE achieves the best resolution for three out of four proteins, with its reconstruction of the pre-60S ribosomal subunit even surpassing the reconstruction

CellPress
OPEN ACCESS

Table 2. Resolution per reconstruction for eight different test datasets, comparing four methods: Blob Picker, Topaz, crYOLO, and cryo-EMMAE, the last three trained on datasets EMPIAR: 10291, 10077

	Without 2D classification (Å)				With 2D classification (Å)				Without 2D classification
Test sets	Blob Picker	Topaz	crYOLO	cryo-EMMAE	Blob Picker	Topaz	crYOLO	cryo-EMMAE	CryoPPP GT
Trained on EMPIAR: 10291									
10028	4.28	–	5.86	**4.13**	4.31	–	6.07	**4.14**	4.22
10081*	5.63	**4.25**	4.61	**4.24**	4.18	3.94	3.97	**3.78**	4.08
10017*	4.56	6.65	>20	**4.55**	4.53	6.33	>20	**4.50**	4.38
11183	6.10	7.89	9.62	**7.03**	6.92	9.24	6.93	**4.65**	7.15
10289*	16.10	6.97	6.49	7.92	7.32	3.77	**3.63**	3.95	8.27
10406	**2.93**	–	3.15	**2.93**	**2.93**	–	3.23	**2.94**	2.97
10077	6.09	–	8.02	7.62	7.46	–	9.28	**6.81**	5.20
Mean	6.77	8.70	7.61	**6.13**	6.20	7.72	6.26	**4.40**	6.11
Rec. Mean	4.35 (4/7)	5.45 (2/7)	5.81 (4/7)	4.58 (5/7)	4.68 (5/7)	4.68 (3/7)	4.77 (5/7)	4.40 (7/7)	4.67 (5/7)
Trained on EMPIAR: 10077									
10028	4.28	**4.12**	4.16	**4.13**	4.31	**4.07**	4.12	4.14	4.22
10081*	5.63	6.90	10.61	**4.56**	4.18	4.36	9.85	**3.94**	4.08
10017*	**4.56**	10.05	>20	4.65	4.53	14.09	17.14	**4.49**	4.38
11183	6.10	7.49	19.19	**6.87**	6.92	7.36	19.42	**4.53**	7.15
10289*	16.10	**9.46**	13.28	9.69	7.32	3.77	7.76	**3.68**	8.27
10406	**2.93**	**2.91**	**2.91**	**2.90**	**2.93**	**2.90**	**2.93**	**2.93**	2.97
10291*	3.96	3.66	8.19	**3.62**	3.65	3.59	8.30	**3.48**	3.43
Mean	5.91	6.36	8.71	**5.20**	5.66	5.15	7.81	**3.88**	4.93
Rec. Mean	4.27 (5/7)	5.76 (6/7)	7.74 (4/7)	5.20 (7/7)	3.92 (5/7)	5.17 (6/7)	6.17 (4/7)	3.88 (7/7)	4.37 (6/7)

Reconstructions were computed both with and without 2D classification on picked particles, from approximately 300 micrographs per dataset, provided by CryoPPP, EMPIAR: 10017 includes only 84 micrographs. Reconstructions that falsely reconstructed the original density map are underlined. For each test dataset, the best reconstruction resolution is highlighted in bold. Reconstructions were also performed using ground truth particles provided by cryoPPP without 2D classification. Symmetry was imposed on datasets EMPIAR: 10081, 10017, 10289, 10291, corresponding to C4, D2, C8, and C8 symmetries, respectively. The Mean row averages resolution values, assigning 10 Å as penalty for failed reconstructions. The Rec. Mean row averages only correct reconstructions and notes the success ratio. GT, ground truth. The best resolutions are highlighted in bold. Asterisks denote imposed symmetries: C4 (EMPIAR: 10081), D2 (EMPIAR: 10017), C8 (EMPIAR: 10289, 10291).

obtained from the ground truth particles. Notably, all four methods struggled to reconstruct the E2 core of the OGDHc at a high resolution, likely due to the low particle count of this protein in the micrographs.

Real case studies

We further conducted a complete 3D reconstruction pipeline for six EMPIAR datasets to provide a direct comparison with the originally reported resolutions. These datasets include a wide variety of structures. For these reconstructions, we used the complete available datasets from the EMPIAR database. The accession numbers are EMPIAR: 10005, 10028, 10049, 10291, 10433, 10955, with corresponding number of micrographs 771, 1,081, 680, 300, 1,280, and 270, respectively; in total 4,382 micrographs were processed and picked.

In Table S2 we report the resolutions, and in Figure S1 we compare the published 3D density maps with those generated using cryo-EMMAE for particle picking. The cryo-EMMAE model used for particle picking was trained on the 20 EMPIAR datasets from cryoPPP, including 180 micrographs from each of the EMPIAR: 10028, 10291 datasets.

For the 3D reconstructions, we imported the particles into CryoSparc v.4.4.0 and performed a single round of 2D classification, then executed *ab initio* reconstruction using the selected particles, followed by homogeneous refinement. When applicable, symmetry was imposed in alignment with the published structures.

Results in Table S2 demonstrate that cryo-EMMAE not only generalizes well to unseen datasets but also achieves resolutions comparable to the published structures, with minimal processing limited to a single round of 2D classification. The mean resolution difference between the published structures and those obtained using cryo-EMMAE is 0.16 Å, with our method even surpassing the published resolution in two cases. Overall, all differences remain within 0.6 Å. An interesting observation is the number of particles used for 3D reconstruction. Both published maps and those with cryo-EMMAE utilize a similar number of particles, except for dataset EMPIAR: 10955, where our method employs a significantly lower number. However, the mean number of particles across all six datasets remains nearly identical.

The maps presented in Figure S1 qualitatively support the reported resolutions, demonstrating a close similarity to the published density maps in EMDB. A noteworthy case is EMPIAR: 10433, which corresponds to the SARS-CoV-2 spike protein. In their analysis, the authors fine-tuned Topaz on the same dataset

Table 3. Resolution and particle counts per reconstruction are presented for the four proteins from the 854-micrograph subset of EMPIAR: 10892

With 2D Classification	3D reconstruction resolution (Å)				No. of particles					
Proteins	Blob Picker	Topaz	crYOLO	cryo-EMMAE	GT	Blob Picker	Topaz	crYOLO	cryo-EMMAE	GT
Pre-60S ribosomal subunit	6.73	5.05	4.44	**4.38**	4.64	8,063	13,877	24,127	28,008	15,670
Fatty acid synthase*	**4.44**	5.73	6.41	5.01	4.43	2,808	1,353	767	1,135	2,567
OGDHc E2 core*	10.41	8.19	9.97	**9.65**	4.05	2,011	742	660	162	1,128
PDHc E2 core*	–	6.86	–	**4.53**	3.96	0	623	0	1,139	3,782
Mean	7.79	6.91	7.70	**5.89**	4.27	4,294	4,149	8,518	10,094	5,787
Rec. Mean	5.59 (2/4)	5.88 (3/4)	6.94 (3/4)	5.89 (4/4)						

Reconstructions for the four methods (Blob Picker, Topaz, crYOLO, and cryo-EMMAE) were computed using two rounds of 2D classification on picked particles, with failed reconstructions underlined. The best resolution per dataset is highlighted in bold. Reconstructions using ground truth particles were also performed. Symmetry was imposed during homogeneous refinement for FAS, OGDHc, and PDHc, corresponding to D3, O, and I symmetries, respectively. The Mean row averages resolution values, assigning a penalty of 10 Å for failed reconstructions, and also computes the average particle count only for correctly reconstructed proteins per method. The Rec. Mean row averages only successful reconstructions and includes the success ratio. GT, ground truth. Best resolution results are shown in bold. Asterisks mark imposed symmetries: D2 (FAS), O (OGDHc), and I (PDHc).

for particle picking. This process required manually selecting particles, training Topaz, and subsequently using it for automated picking.[34] In contrast, we applied cryo-EMMAE directly on the micrographs of the dataset without any fine-tuning or prior relationship between the initial training set of cryo-EMMAE and EMPIAR: 10433, resulting in a highly similar structure both qualitatively and in terms of resolution.

DISCUSSION

In this work we present cryo-EMMAE, the first self-supervised method applied to the highly complex cryo-EM data. A MAE is initially trained to reconstruct patches of the initial micrographs. Multiple levels of clustering are then applied on the representation space of MAE in order to hierarchically denoise the micrographs. The final denoised micrograph consists of a segmentation of the particles from the background.

Our experimental evaluation shows that cryo-EMMAE exhibits stable generalization capabilities when applied to unseen data, significantly outperforming supervised methods. This suggests that our approach effectively reduces the impact of dataset-specific noise and the inherent characteristics of micrographs. These results imply that cryo-EMMAE is able to learn the necessary invariances, regardless of the experimental variances that are common in cryo-EM procedures.

Notably, this generalization ability is maintained even when trained with only a small number of micrographs. We hypothesize that this behavior stems from the standardization introduced during the micrograph normalization process in our pre-processing. This standardization helps reduce the influence of experimental factors, such as ice thickness, on micrograph noise. As a result, the noise is normalized, making the data less variable at a local scale. Additionally, reconstructing random patches during training MAE further enhances the model's robustness to such experimental inconsistencies.

It is important to note that Topaz[23] shows low precision, even when tested on the same dataset it was trained on. Particle-picking models generally prioritize recall, as they are often used to assist lab practitioners in quickly annotating data.[23] In this context, high recall is more critical, as annotators can manually filter out false positives through 2D classification. However, for fully automated systems, good precision is essential to ensure accuracy and minimize errors without the need for manual intervention. In contrast, cryo-EMMAE, with its strong generalization capabilities and stable precision, shows promise for improving automation in such tasks. Additionally, we demonstrate that, as the number of clusters increases, precision improves while recall decreases. This suggests that the clustering process filters out more background pixels, but also removes some true particle positions. In our method, by selecting the optimal number of clusters, users can balance precision and recall according to their specific needs.

The superior generalization performance is also evident in the resolution of 3D reconstructions across the eight different test datasets, comparing four methods and the annotated particle sets from cryoPPP.[33] cryo-EMMAE significantly outperforms the other two machine learning approaches and achieves mean resolution approximately 0.7 and 1.8 Å better than CryoSPARC's Blob Picker, without and with 2D classification, respectively.

The results of CryoPPP particles, highlight the inherent biases of annotated datasets, which often fail to provide the optimal set of particles needed to achieve the best possible resolution for a given dataset of micrographs. This observation is apparent in two key comparisons within our work: (1) the major differences between the metrics and the reconstruction evaluations of the methods, where the superiority of cryo-EMMAE becomes more evident on reconstructions studies and (2) the advantage of cryo-EMMAE's reconstructions compared with the CryoPPP's annotated particles. Consequently, our work underlines the importance of evaluating cryo-EM particle-picking methods based on the resolution of the resulting 3D density map. This objective should also be the end-to-end goal of any particle-picking machine learning methodology.

Finally, cryo-EMMAE demonstrated its strength in practical and demanding scenarios, excelling in multi-particle micrographs. It outperformed all the methods compared and successfully reconstructed correctly all four proteins from the original

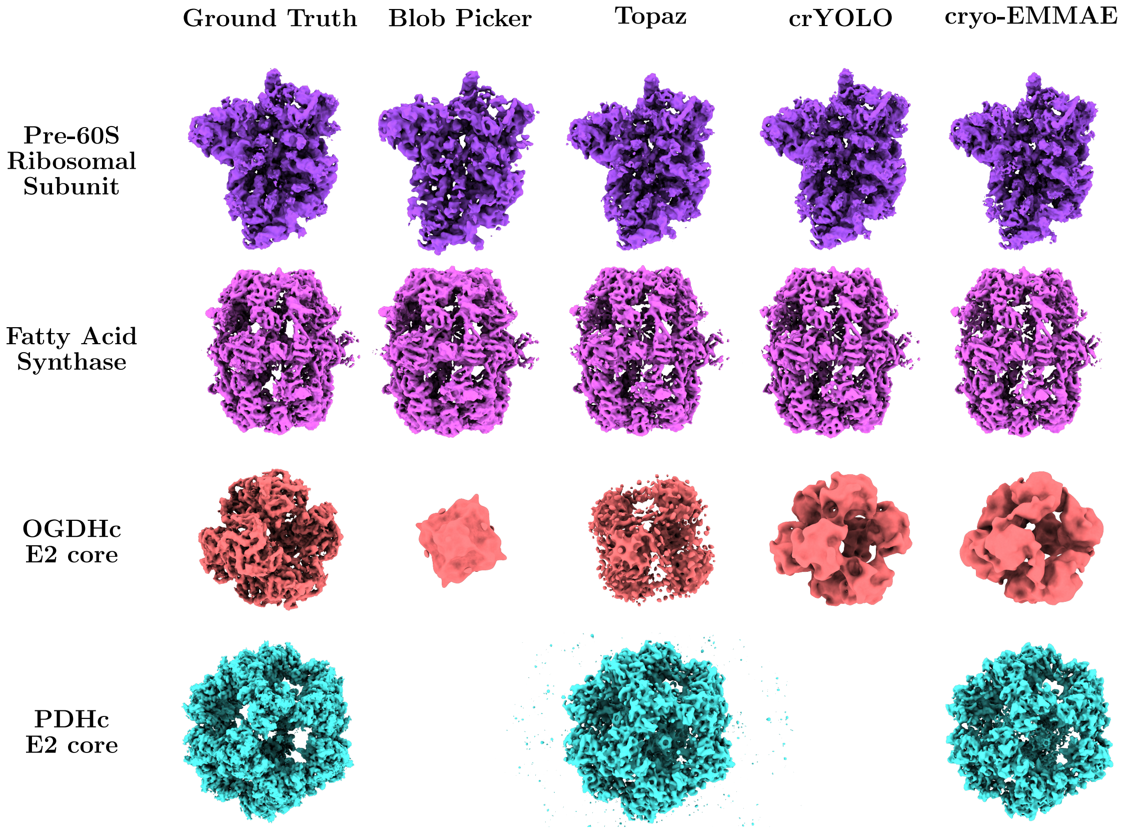

Figure 4. Visualized reconstructed density maps of the four proteins from EMPIAR: 10892 are shown for ground truth and the four tested methods

Each protein in the dataset is represented by a distinct color. The learning-based methods (Topaz, crYOLO, and cryo-EMMAE) were trained using 20 different EMPIAR datasets. The volumes reconstructed by the four methods (Blob Picker, Topaz, crYOLO, and cryo-EMMAE) include two 2D classification-class selection steps, whereas the reconstructions using ground truth particles from the original study do not incorporate this step. Density values for all maps range from −1 to 3, with thresholds applied close to 1.

study. Notably, cryo-EMMAE surpassed the second-best method (Topaz) by more than 1 Å of mean resolution and even managed to surpass the resolution of ground truth particles for the pre-60S ribosomal subunit. Overall, our work helps reduce the heavy dependence on costly expert annotations—which are often not optimal—for cryo-EM data analysis, paving the way for more cost-effective and automated solutions in the field of cryo-EM analysis.

Related work

Cryo-EM particle localization is a crucial step in cryo-EM particle analysis. This is apparent considering the variety of approaches that have been proposed. These approaches range from traditional computer vision techniques to more advanced machine and deep learning methods. Traditional methods are generally categorized into two main approaches. The first, known as the template-based methods,[10,11] relies on projections of a given protein structure to match in experimental micrographs. While this method provides a solid framework, it introduces human bias through the selection of the template protein, potentially losing different projections and conformations, or even leading to the Einstein-from-noise effect.[35–37] On the other hand, template-free approaches offer more flexibility and ease of implementation. Techniques such as Laplacian of Gaussian,[12,13] Difference of Gaussians,[14] and Blob Picker[15] are among the most commonly used. Despite their ease of use, these methods often prioritize picking as many particles as possible, which leads to high false-positive rates. These inaccuracies can negatively impact subsequent analysis steps, increasing processing times and potentially introducing noise into the final reconstructed volume.

Deep learning methods have shown success in particle picking. All methods in the literature to date rely on annotated datasets, falling under the supervised learning scheme. These methods address particle picking from three primary perspectives, predominantly employing convolutional neural networks.[38] First, binary classification of micrograph windows as either containing particles or not has been implemented by methods such as DeepEM,[22] Topaz,[23] Warp,[24] and DeepCryoPicker.[26] Second, segmentation of micrographs into background and particle classes has been addressed by methodologies such as DeepPicker,[17] Pixer,[18] PARSED,[19] DRPnet,[20] and CryoSegNet.[21] Finally, for object detection, a less common yet fitting approach, methodologies such as crYOLO,[28] EPicker,[29] and CryoTransformer[30] have been developed.

The methods that are mostly used in practice are Topaz[23] and crYOLO[28] in which researchers typically manually annotate a small number of experimental micrographs with particles to refine pretrained models. The fine-tuned models are then used to predict particles in experimental micrographs. However, this process is time-consuming and relies on experienced annotators, who may introduce bias during the selection of particles. Previous studies have observed that crYOLO[28] often misses true protein particles, while Topaz[23] picks a lot of duplicate particles and false positives.[21,27,29,30]

Future work and broader impact

From a technical perspective, cryo-EMMAE introduces an effective method for representation learning on images with extremely low signal/noise ratios, coined specifically for segmentation tasks. This approach can be extended to various challenges in other scientific domains that share similar characteristics. For instance, our pipeline can be easily adapted to other biomedical image segmentation tasks, such as localizing protein complexes within cells or organelles using cryoelectron tomography, performing multipurpose segmentation (e.g., multi-organ, multi-disease, and multi-phase) with computed tomography and magnetic resonance imaging, localizing cells in microscopy data, or detecting and segmenting pathological features in histopathology,[39–43] among others. Although certain parameters, such as image patching, number of clustering centers, and post-processing steps, would require further hyperparameter search to be in line to the characteristics of the target domain, the overall pipeline of the methodology remains unchanged.

A potential extension, inspired by cryo-EMMAE could be the integration of particle picking and 3D reconstruction steps into an end-to-end self-supervised framework. This coupling has the potential to transform cryo-EM analysis by reducing the dependence on extensive expert supervision and facilitating a more efficient, higher-throughput unsupervised workflow. Consequently, researchers could focus on more critical tasks, such as improving experimental protocols and interpreting results, instead of the labor-intensive data analysis.

From a broader perspective, the automation of cryo-EM analysis can enhance scientific research across four key areas: (1) accelerating the determination of high-resolution protein structures, (2) advancing our understanding of disease mechanisms and promoting drug discovery, (3) helping healthcare innovation through personalized medicine and faster vaccine designing, and (4) influencing cross-disciplinary field with similar computational needs, such as nanotechnology and material science.

Our work hopefully contributes to these advancements by pushing forward automation in cryo-EM data analysis.

Limitations of the study

While cryo-EMMAE demonstrates strong performance across unseen cryo-EM datasets, certain limitations remain. Our method struggles with highly crowded micrographs, low contrast, and large particles, situations where accurately segmenting individual particles, in general, becomes more challenging; these difficulties are universal to other literature methods.

Furthermore, increasing the amount of training data for cryo-EMMAE has shown limited impact on improving performance, suggesting that cryo-EMMAE's scalability with larger datasets needs further investigation; one potential reason for that limitation might be the preprocessing steps, which remove a significant amount of high-frequency information from the micrographs. As a result, our method focus on the low-frequency components—common across all micrographs—which might contribute to its stable performance across the test datasets. However, this focus on low frequencies may limit its ability to learn more highly variable characteristics of the data.

The primary goal of our empirical evaluations was to assess the generalization capabilities of all models, specifically to determine if they can be applied directly to unannotated laboratory data to accelerate structural biology research. However, in cases where annotated data are available, supervised methods may outperform our approach on those specific datasets. Our study is limited in this regard, as it does not evaluate performance under these conditions.

We further did not perform ablation studies to evaluate the impact of our preprocessing pipeline on the supervised methods. We speculate that incorporating this preprocessing would improve their absolute performance, although not necessarily their generalization ability. Instead, we chose to reproduce their original methodology, where this preprocessing step is not included.

RESOURCE AVAILABILITY

Lead contact

Requests for further information and resources should be directed to and will be fulfilled by the lead contact, Andreas Zamanos (a.zamanos@di.uoa.gr).

Materials availability

This study did not generate unique reagents.

Data and code availability

- The data used in this study are archived on Zenodo: https://zenodo.org/records/11659477. The source dataset for CryoPPP can be downloaded by following the instructions at: https://github.com/BioinfoMachineLearning/cryoppp. All experimental data are also accessible via the EMPIAR database: https://www.ebi.ac.uk/empiar/.
- Our source code is available at GitHub: https://github.com/azamanos/Cryo-EMMAE. An archival DOI is listed in the key resources table.
- Any additional information required to reanalyze the data reported in this paper is available from the lead contact upon request.

ACKNOWLEDGMENTS

A.Z., G.B., and Y.P. were supported by project MIS 5154714 of the National Recovery and Resilience Plan Greece 2.0 funded by the European Union under the NextGenerationEU Program. P.K. was supported by the Hellenic Foundation for Research and Innovation (HFRI) under the 4th Call for HFRI PhD Fellowships (Fellowship no. 10816). P.L.K. was supported by the European Union through funding of the Horizon Europe ERA Chair "hot4cryo" project no. 101086665, the Federal Ministry of Education and Research (BMBF, ZIK program) (grant nos. 03Z22HN23, 03Z22HI2, and 03COV04), the European Regional Development Funds (EFRE) for Saxony-Anhalt (grant no. ZS/2016/04/78115), the Deutsche Forschungsgemeinschaft (project nos. 391498659, RTG 2467 and 514901783, SFB 1664 [A04, C04, and D01]), and the Martin Luther University Halle-Wittenberg. The authors would like to thank Dr. Fotis L. Kyrilis for his valuable insights into the procedures of 3D reconstructions. Computational resources were granted with the support of GRNET.

AUTHOR CONTRIBUTIONS

Conceptualization, A.Z. and Y.P.; methodology, A.Z., P.K., and Y.P.; investigation, A.Z., P.K., G.B., and Y.P.; writing – original draft, A.Z., P.K., G.B., and Y.P.; writing – review & editing, A.Z., P.K., G.B., P.L.K., and Y.P.; funding acquisition, P.L.K. and Y.P.; resources, P.L.K. and Y.P.; supervision, P.L.K. and Y.P.

DECLARATION OF INTERESTS

The authors declare no competing interests.

STAR★METHODS

Detailed methods are provided in the online version of this paper and include the following:

- KEY RESOURCES TABLE
- EXPERIMENTAL MODEL AND STUDY PARTICIPANT DETAILS
- METHOD DETAILS
  - Micrograph preprocessing
  - Representation learning
  - Implementation details
  - Inference
  - Experimental set-up
  - 3D reconstruction methodology
- QUANTIFICATION AND STATISTICAL ANALYSIS

SUPPLEMENTAL INFORMATION

Supplemental information can be found online at https://doi.org/10.1016/j.crmeth.2025.101089.

Received: December 24, 2024
Revised: February 25, 2025
Accepted: June 10, 2025
Published: July 7, 2025

REFERENCES

1. Milne, J.L.S., Borgnia, M.J., Bartesaghi, A., Tran, E.E.H., Earl, L.A., Schauder, D.M., Lengyel, J., Pierson, J., Patwardhan, A., and Subramaniam, S. (2013). Cryo-electron microscopy–a primer for the non-microscopist. FEBS J. *280*, 28–45. https://doi.org/10.1111/febs.12078.
2. Nogales, E., and Scheres, S.H.W. (2015). Cryo-em: a unique tool for the visualization of macromolecular complexity. Mol. Cell *58*, 677–689. https://doi.org/10.1016/j.molcel.2015.02.019.
3. Doerr, A. (2016). Single-particle cryo-electron microscopy: a brief overview of how to solve a macromolecular structure using single-particle cryo-electron microscopy (cryo-em). Nat. Methods *13*, 23–24. https://doi.org/10.1038/nmeth.3700.
4. Bendory, T., Bartesaghi, A., and Singer, A. (2020). Single-particle cryo-electron microscopy: Mathematical theory, computational challenges, and opportunities. IEEE Signal Process. Mag. *37*, 58–76. https://doi.org/10.1109/MSP.2019.2957822.
5. Wu, J.-G., Yan, Y., Zhang, D.-X., Liu, B.-W., Zheng, Q.-B., Xie, X.-L., Liu, S.-Q., Ge, S.-X., Hou, Z.-G., and Xia, N.-S. (2022). Machine learning for structure determination in single-particle cryo-electron microscopy: A systematic review. IEEE Trans. Neural Netw. Learn. Syst. *33*, 452–472. https://doi.org/10.1109/TNNLS.2021.3131325.
6. Chung, J.M., Durie, C.L., and Lee, J. (2022). Artificial intelligence in cryo-electron microscopy. Life *12*, 1267. https://doi.org/10.3390/life12081267.
7. Agard, D., Cheng, Y., Glaeser, R.M., and Subramaniam, S. (2014). Single-particle cryo-electron microscopy (cryo-em): Progress, challenges, and perspectives for further improvement. Adv. Imaging Electron Phys. *185*, 113–137. https://doi.org/10.1016/B978-0-12-800144-8.00002-1.
8. Baxter, W.T., Grassucci, R.A., Gao, H., and Frank, J. (2009). Determination of signal-to-noise ratios and spectral snrs in cryo-em low-dose imaging of molecules. J. Struct. Biol. *166*, 126–132. https://doi.org/10.1016/j.jsb.2009.02.012.
9. Skalidis, I., Kyrilis, F.L., Tüting, C., Hamdi, F., Chojnowski, G., and Kastritis, P.L. (2022). Cryo-em and artificial intelligence visualize endogenous protein community members. Struct *30*, 575–589. https://doi.org/10.1016/j.str.2022.01.001.
10. Scheres, S.H.W. (2012). Relion: implementation of a bayesian approach to cryo-em structure determination. J. Struct. Biol. *180*, 519–530. https://doi.org/10.1016/j.jsb.2012.09.006.
11. Scheres, S.H.W. (2015). Semi-automated selection of cryo-em particles in relion-1.3. J. Struct. Biol. *189*, 114–122. https://doi.org/10.1016/j.jsb.2014.11.010.
12. Woolford, D., Hankamer, B., and Ericksson, G. (2007). The laplacian of gaussian and arbitrary z-crossings approach applied to automated single particle reconstruction. J. Struct. Biol. *159*, 122–134. https://doi.org/10.1016/j.jsb.2007.03.003.
13. Woolford, D., Ericksson, G., Rothnagel, R., Muller, D., Landsberg, M.J., Pantelic, R.S., McDowall, A., Pailthorpe, B., Young, P.R., Hankamer, B., and Banks, J. (2007). Swarmps: rapid, semi-automated single particle selection software. J. Struct. Biol. *157*, 174–188. https://doi.org/10.1016/j.jsb.2006.04.006.
14. Voss, N.R., Yoshioka, C.K., Radermacher, M., Potter, C.S., and Carragher, B. (2009). Dog picker and tiltpicker: software tools to facilitate particle selection in single particle electron microscopy. J. Struct. Biol. *166*, 205–213. https://doi.org/10.1016/j.jsb.2009.01.004.
15. Punjani, A., Rubinstein, J.L., Fleet, D.J., and Brubaker, M.A. (2017). cryosparc: algorithms for rapid unsupervised cryo-em structure determination. Nat. Methods *14*, 290–296. https://doi.org/10.1038/nmeth.4169.
16. Al-Azzawi, A., Ouadou, A., Tanner, J.J., and Cheng, J. (2019). Autocryopicker: an unsupervised learning approach for fully automated single particle picking in cryo-em images. BMC Bioinform *20*, 326. https://doi.org/10.1186/s12859-019-2926-y.
17. Wang, F., Gong, H., Liu, G., Li, M., Yan, C., Xia, T., Li, X., and Zeng, J. (2016). Deeppicker: A deep learning approach for fully automated particle picking in cryo-em. J. Struct. Biol. *195*, 325–336. https://doi.org/10.1016/j.jsb.2016.07.006.
18. Zhang, J., Wang, Z., Chen, Y., Han, R., Liu, Z., Sun, F., and Zhang, F. (2019). Pixer: an automated particle-selection method based on segmentation using a deep neural network. BMC Bioinform *20*, 41. https://doi.org/10.1186/s12859-019-2614-y.
19. Yao, R., Qian, J., and Huang, Q. (2020). Deep-learning with synthetic data enables automated picking of cryo-em particle images of biological macromolecules. Bioinformatics *36*, 1252–1259. https://doi.org/10.1093/bioinformatics/btz728.
20. Nguyen, N.P., Ersoy, I., Gotberg, J., Bunyak, F., and White, T.A. (2021). Drpnet: automated particle picking in cryo-electron micrographs using deep regression. BMC Bioinform *22*, 55. https://doi.org/10.1186/s12859-020-03948-x.
21. Gyawali, R., Dhakal, A., Wang, L., and Cheng, J. (2024). Cryosegnet: accurate cryo-em protein particle picking by integrating the foundational ai image segmentation model and attention-gated u-net. Preprint at bioRxiv *25*. https://doi.org/10.1101/2023.10.02.560572.
22. Zhu, Y., Ouyang, Q., and Mao, Y. (2017). A deep convolutional neural network approach to single-particle recognition in cryo-electron microscopy. BMC Bioinform *18*, 348. https://doi.org/10.1186/s12859-017-1757-y.
23. Bepler, T., Morin, A., Rapp, M., Brasch, J., Shapiro, L., Noble, A.J., and Berger, B. (2019). Positive-unlabeled convolutional neural networks for particle picking in cryo-electron micrographs. Nat. Methods *16*, 1153–1160. https://doi.org/10.1038/s41592-019-0575-8.

24. Tegunov, D., and Cramer, P. (2019). Real-time cryo-electron microscopy data preprocessing with warp. Nat. Methods *16*, 1146–1152. https://doi.org/10.1038/s41592-019-0580-y.

25. McSweeney, D.M., McSweeney, S.M., and Liu, Q. (2020). A self-supervised workflow for particle picking in cryo-em. IUCrJ *7*, 719–727. https://doi.org/10.1107/S2052252520007241.

26. Al-Azzawi, A., Ouadou, A., Max, H., Duan, Y., Tanner, J.J., and Cheng, J. (2020). Deepcryopicker: fully automated deep neural network for single protein particle picking in cryo-em. BMC Bioinform *21*, 509–538. https://doi.org/10.1186/s12859-020-03809-7.

27. Xu, C., Zhan, X., and Xu, M. (2024). Cryomae: Few-shot cryo-em particle picking with masked autoencoders. Preprint at arXiv. https://doi.org/10.48550/arXiv.2404.10178.

28. Wagner, T., Merino, F., Stabrin, M., Moriya, T., Antoni, C., Apelbaum, A., Hagel, P., Sitsel, O., Raisch, T., Prumbaum, D., et al. (2019). Sphire-cryolo is a fast and accurate fully automated particle picker for cryo-em. Commun. Biol. *2*, 218. https://doi.org/10.1038/s42003-019-0437-z.

29. Zhang, X., Zhao, T., Chen, J., Shen, Y., and Li, X. (2022). Epicker is an exemplar-based continual learning approach for knowledge accumulation in cryoem particle picking. Nat. Commun. *13*, 2468. https://doi.org/10.1038/s41467-022-29994-y.

30. Dhakal, A., Gyawali, R., Wang, L., and Cheng, J. (2024). Cryotransformer: a transformer model for picking protein particles from cryo-em micrographs. Bioinformatics *40*, btae109. https://doi.org/10.1093/bioinformatics/btae109.

31. Kyrilis, F.L., Meister, A., and Kastritis, P.L. (2019). Integrative biology of native cell extracts: a new era for structural characterization of life processes. Biol. Chem. *400*, 831–846. https://doi.org/10.1515/hsz-2018-0445.

32. Kyrilis, F.L., Semchonok, D.A., Skalidis, I., Tüting, C., Hamdi, F., O'Reilly, F.J., Rappsilber, J., and Kastritis, P.L. (2021). Integrative structure of a 10-megadalton eukaryotic pyruvate dehydrogenase complex from native cell extracts. Cell Rep. *34*, 108727. https://doi.org/10.1016/j.celrep.2021.108727.

33. Dhakal, A., Gyawali, R., Wang, L., and Cheng, J. (2023). A large expert-curated cryo-em image dataset for machine learning protein particle picking. Sci. Data *10*, 392. https://doi.org/10.1038/s41597-023-02280-2.

34. Herrera, N.G., Morano, N.C., Celikgil, A., Georgiev, G.I., Malonis, R.J., Lee, J.H., Tong, K., Vergnolle, O., Massimi, A.B., Yen, L.Y., et al. (2020). Characterization of the sars-cov-2 s protein: biophysical, biochemical, structural, and antigenic analysis. Preprint at bioRxiv *6*. https://doi.org/10.1101/2020.06.14.150607.

35. Henderson, R. (2013). Avoiding the pitfalls of single particle cryo-electron microscopy: Einstein from noise. Proc. Natl. Acad. Sci. USA *110*, 18037–18041. https://doi.org/10.1073/pnas.1314449110.

36. van Heel, M. (2013). Finding trimeric hiv-1 envelope glycoproteins in random noise. Proc. Natl. Acad. Sci. USA *110*, E4175–E4177. https://doi.org/10.1073/pnas.1314353110.

37. Subramaniam, S. (2013). Structure of trimeric hiv-1 envelope glycoproteins. Proc. Natl. Acad. Sci. USA *110*, E4172–E4174. https://doi.org/10.1073/pnas.1313802110.

38. Gu, J., Wang, Z., Kuen, J., Ma, L., Shahroudy, A., Shuai, B., Liu, T., Wang, X., Wang, G., Cai, J., and Chen, T. (2018). Recent advances in convolutional neural networks. Pattern Recognit *77*, 354–377. https://doi.org/10.1016/j.patcog.2017.10.013.

39. Litjens, G., Toth, R., Van De Ven, W., Hoeks, C., Kerkstra, S., Van Ginneken, B., Vincent, G., Guillard, G., Birbeck, N., Zhang, J., et al. (2014). Evaluation of prostate segmentation algorithms for mri: the promise12 challenge. Med. Image Anal. *18*, 359–373. https://doi.org/10.1016/j.media.2013.12.002.

40. Simpson, A.L., Antonelli, M., Bakas, S., Bilello, M., Farahani, K., Van Ginneken, B., Kopp-Schneider, A., Landman, B.A., Litjens, G., Menze, B., et al. (2019). A large annotated medical image dataset for the development and evaluation of segmentation algorithms. Preprint at arXiv. https://doi.org/10.48550/arXiv.1902.09063.

41. Edlund, C., Jackson, T.R., Khalid, N., Bevan, N., Dale, T., Dengel, A., Ahmed, S., Trygg, J., and Sjögren, R. (2021). Livecell—a large-scale dataset for label-free live cell segmentation. Nat. Methods *18*, 1038–1045. https://doi.org/10.1038/s41592-021-01249-6.

42. Da, Q., Huang, X., Li, Z., Zuo, Y., Zhang, C., Liu, J., Chen, W., Li, J., Xu, D., Hu, Z., et al. (2022). Digestpath: A benchmark dataset with challenge review for the pathological detection and segmentation of digestive-system. Med. Image Anal. *80*, 102485. https://doi.org/10.1016/j.media.2022.102485.

43. Ji, Y., Bai, H., Ge, C., Yang, J., Zhu, Y., Zhang, R., Li, Z., Zhanng, L., Ma, W., Wan, X., et al. (2022). Amos: A large-scale abdominal multi-organ benchmark for versatile medical image segmentation. Adv. Neural Inf. Process. Syst. *35*, 36722–36732. https://doi.org/10.48550/arXiv.2206.08023.

44. Burendei, B., Shinozaki, R., Watanabe, M., Terada, T., Tani, K., Fujiyoshi, Y., and Oshima, A. (2020). Cryo-em structures of undocked innexin-6 hemichannels in phospholipids. Sci. Adv. *6*, eaax3157. https://doi.org/10.1126/sciadv.aax3157.

45. Fischer, N., Neumann, P., Bock, L.V., Maracci, C., Wang, Z., Paleskava, A., Konevega, A.L., Schröder, G.F., Grubmüller, H., Ficner, R., et al. (2016). The pathway to gtpase activation of elongation factor selb on the ribosome. Nature *540*, 80–85. https://doi.org/10.1038/nature20560.

46. Mashtalir, N., Suzuki, H., Farrell, D.P., Sankar, A., Luo, J., Filipovski, M., D'Avino, A.R., St Pierre, R., Valencia, A.M., Onikubo, T., et al. (2020). A structural model of the endogenous human baf complex informs disease mechanisms. Cell *183*, 802–817.e24. https://doi.org/10.1016/j.cell.2020.09.051.

47. Oldham, M.L., Grigorieff, N., and Chen, J. (2016). Structure of the transporter associated with antigen processing trapped by herpes simplex virus. eLife *5*, e21829. https://doi.org/10.7554/eLife.21829.

48. Wong, W., Bai, X.-c., Brown, A., Fernandez, I.S., Hanssen, E., Condron, M., Tan, Y.H., Baum, J., and Scheres, S.H.W. (2014). Cryo-em structure of the plasmodium falciparum 80s ribosome bound to the anti-protozoan drug emetine. eLife *3*, e03080. https://doi.org/10.7554/eLife.03080.

49. Lee, C.-H., and MacKinnon, R. (2017). Structures of the human hcn1 hyperpolarization-activated channel. Cell *168*, 111–120. https://doi.org/10.1016/j.cell.2016.12.023.

50. Tan, Y.Z., Baldwin, P.R., Davis, J.H., Williamson, J.R., Potter, C.S., Carragher, B., and Lyumkis, D. (2017). Addressing preferred specimen orientation in single-particle cryo-em through tilting. Nat. Methods *14*, 793–796. https://doi.org/10.1038/nmeth.4347.

51. Falzone, M.E., Rheinberger, J., Lee, B.-C., Peyear, T., Sasset, L., Raczkowski, A.M., Eng, E.T., Di Lorenzo, A., Andersen, O.S., Nimigean, C.M., and Accardi, A. (2019). Structural basis of ca2+-dependent activation and lipid transport by a tmem16 scramblase. eLife *8*, e43229. https://doi.org/10.7554/eLife.43229.

52. Nicholson, D., Edwards, T.A., O'Neill, A.J., and Ranson, N.A. (2020). Structure of the 70s ribosome from the human pathogen acinetobacter baumannii in complex with clinically relevant antibiotics. Struct *28*, 1087–1100. https://doi.org/10.1016/j.str.2020.08.004.

53. Li, J., Han, L., Vallese, F., Ding, Z., Choi, S.K., Hong, S., Luo, Y., Liu, B., Chan, C.K., Tajkhorshid, E., et al. (2021). Cryo-em structures of escherichia coli cytochrome bo 3 reveal bound phospholipids and ubiquinone-8 in a dynamic substrate binding site. Proc. Natl. Acad. Sci. USA *118*, e2106750118. https://doi.org/10.1073/pnas.2106750118.

54. Gao, Y., Cao, E., Julius, D., and Cheng, Y. (2016). Trpv1 structures in nanodiscs reveal mechanisms of ligand and lipid action. Nature *534*, 347–351. https://doi.org/10.1038/nature17964.

55. Liu, Y., Cao, C., Huang, X.-P., Gumpper, R.H., Rachman, M.M., Shih, S.-L., Krumm, B.E., Zhang, S., Shoichet, B.K., Fay, J.F., and Roth, B.L. (2023). Ligand recognition and allosteric modulation of the human mrgprx1

receptor. Nat. Chem. Biol. *19*, 416–422. https://doi.org/10.1038/s41589-022-01173-6.

56. Kuzuya, M., Hirano, H., Hayashida, K., Watanabe, M., Kobayashi, K., Terada, T., Mahmood, M.I., Tama, F., Tani, K., Fujiyoshi, Y., and Oshima, A. (2022). Structures of human pannexin-1 in nanodiscs reveal gating mediated by dynamic movement of the n terminus and phospholipids. Sci. Signal. *15*, eabg6941. https://doi.org/10.1126/scisignal.abg6941.

57. Bartesaghi, A., Merk, A., Banerjee, S., Matthies, D., Wu, X., Milne, J.L.S., and Subramaniam, S. (2015). 2.2 å resolution cryo-em structure of β--galactosidase in complex with a cell-permeant inhibitor. Science *348*, 1147–1151. https://doi.org/10.1126/science.aab1576.

58. Koning, R.I., Gomez-Blanco, J., Akopjana, I., Vargas, J., Kazaks, A., Tars, K., Carazo, J.M., and Koster, A.J. (2016). Asymmetric cryo-em reconstruction of phage ms2 reveals genome structure in situ. Nat. Commun. *7*, 12524. https://doi.org/10.1038/ncomms12524.

59. Kim, L.Y., Rice, W.J., Eng, E.T., Kopylov, M., Cheng, A., Raczkowski, A.M., Jordan, K.D., Bobe, D., Potter, C.S., and Carragher, B. (2018). Benchmarking cryo-em single particle analysis workflow. Front. Mol. Biosci. *5*, 50. https://doi.org/10.3389/fmolb.2018.00050.

60. Tan, Y.Z., and Rubinstein, J.L. (2020). Through-grid wicking enables high-speed cryoem specimen preparation. Acta Crystallogr. D Struct. Biol. *76*, 1092–1103. https://doi.org/10.1107/S2059798320012474.

61. Dong, Y., Zhang, S., Wu, Z., Li, X., Wang, W.L., Zhu, Y., Stoilova-McPhie, S., Lu, Y., Finley, D., and Mao, Y. (2019). Cryo-em structures and dynamics of substrate-engaged human 26s proteasome. Nature *565*, 49–55. https://doi.org/10.1038/s41586-018-0736-4.

62. Meng, E.C., Goddard, T.D., Pettersen, E.F., Couch, G.S., Pearson, Z.J., Morris, J.H., and Ferrin, T.E. (2023). Ucsf chimerax: Tools for structure building and analysis. Protein Sci. *32*, e4792. https://doi.org/10.1002/pro.4792.

63. Chen, T., Kornblith, S., Norouzi, M., and Hinton, G. (2020). A Simple Framework for Contrastive Learning of Visual Representations. Preprint at arXiv. https://doi.org/10.48550/arXiv.2002.05709.

64. Caron, M., Touvron, H., Misra, I., Jégou, H., Mairal, J., Bojanowski, P., and Joulin, A. (2021). Emerging properties in self-supervised vision transformers. 2021 IEEE/CVF International Conference on Computer Vision (ICCV). https://doi.org/10.1109/ICCV48922.2021.00951.

65. Zbontar, J., Jing, L., Misra, I., LeCun, Y., and Deny, S. (2021). Barlow Twins: Self-Supervised Learning via Redundancy Reduction. Preprint at arXiv. https://doi.org/10.48550/arXiv.2103.03230.

66. He, K., Chen, X., Xie, S., Li, Y., Dollár, P., and Girshick, R. (2022). Masked autoencoders are scalable vision learners. 2022 IEEE/CVF Conference on Computer Vision and Pattern Recognition (CVPR). https://doi.org/10.1109/CVPR52688.2022.01553.

67. Dosovitskiy, A., Beyer, L., Kolesnikov, A., Weissenborn, D., Zhai, X., Unterthiner, T., Dehghani, M., Minderer, M., Heigold, G., Gelly, S., et al. (2020). An image is worth 16x16 words: Transformers for image recognition at scale. Preprint at arXiv. https://doi.org/10.48550/arXiv.2010.11929.

STAR★METHODS

KEY RESOURCES TABLE

REAGENT or RESOURCE	SOURCE	IDENTIFIER
**Deposited Data**		
EMPIAR: 10291	Burendei et al.[44]	https://www.ebi.ac.uk/empiar/EMPIAR-10291/
EMPIAR: 10077	Fischer et al.[45]	https://www.ebi.ac.uk/empiar/EMPIAR-10077/
EMPIAR: 10590	Mashtalir et al.[46]	https://www.ebi.ac.uk/empiar/EMPIAR-10590/
EMPIAR: 10816	Oldham et al.[47]	https://www.ebi.ac.uk/empiar/EMPIAR-10816/
EMPIAR: 10028	Wong et al.[48]	https://www.ebi.ac.uk/empiar/EMPIAR-10028/
EMPIAR: 10081	Lee et al.[49]	https://www.ebi.ac.uk/empiar/EMPIAR-10081/
EMPIAR: 10096	Tan et al.[50]	https://www.ebi.ac.uk/empiar/EMPIAR-10096/
EMPIAR: 10240	Falzone et al.[51]	https://www.ebi.ac.uk/empiar/EMPIAR-10240/
EMPIAR: 10406	Nicholson et al.[52]	https://www.ebi.ac.uk/empiar/EMPIAR-10406/
EMPIAR: 10289	Burendei et al.[44]	https://www.ebi.ac.uk/empiar/EMPIAR-10289/
EMPIAR: 10737	Li et al.[53]	https://www.ebi.ac.uk/empiar/EMPIAR-10737/
EMPIAR: 10059	Gao et al.[54]	https://www.ebi.ac.uk/empiar/EMPIAR-10059/
EMPIAR: 11183	Liu et al.[55]	https://www.ebi.ac.uk/empiar/EMPIAR-11183/
EMPIAR: 10017	Scheres[11]	https://www.ebi.ac.uk/empiar/EMPIAR-10017/
EMPIAR: 10760	Kuzuya et al.[56]	https://www.ebi.ac.uk/empiar/EMPIAR-10760/
EMPIAR: 10061	Bartesaghi et al.[57]	https://www.ebi.ac.uk/empiar/EMPIAR-10061/
EMPIAR: 10075	Koning et al.[58]	https://www.ebi.ac.uk/empiar/EMPIAR-10075/
EMPIAR: 10184	Kim et al.[59]	https://www.ebi.ac.uk/empiar/EMPIAR-10184/
EMPIAR: 10532	Tan and Rubinstein[60]	https://www.ebi.ac.uk/empiar/EMPIAR-10532/
EMPIAR: 10669	Dong et al.[61]	https://www.ebi.ac.uk/empiar/EMPIAR-10669/
CryoPPP annotated dataset	Dhakal et al.[33]	https://calla.rnet.missouri.edu/cryoppp/
EMPIAR: 10892 (cell extracts dataset)	Skalidis et al.[9]	https://www.ebi.ac.uk/empiar/EMPIAR-10892/
**Software and Algorithms**		
cryo-EMMAE	This study	https://doi.org/10.5281/zenodo.15542966
Topaz	Bepler et al.[23]	https://github.com/tbepler/topaz
crYOLO	Wagner et al.[28]	https://cryolo.readthedocs.io/
Blob Picker	Punjani et al.[15]	https://cryosparc.com/
ChimeraX v.1.8	Meng et al.[62]	https://www.cgl.ucsf.edu/chimerax/
CryoSPARC v.4.4.0	Punjani et al.[15]	https://cryosparc.com/

EXPERIMENTAL MODEL AND STUDY PARTICIPANT DETAILS

This study did not use experimental models commonly employed in life sciences.

METHOD DETAILS

Micrograph preprocessing

Protein projections within cryo-EM micrographs are essentially 2D representations of the protein under investigation; it is necessary for these projections to capture high-frequency details for revealing details of the protein's 3D structure. However, these images suffer from varying degrees of high-frequency noise, obscuring the structural clarity of the data. Therefore, it is vital to devise a filtering process, to enhance the distinction of such information during picking.

To standardize the background noise in micrographs, we employ a normalization technique outlined in[11] that results in a zero-mean and unit standard deviation noise by adjusting for noise variations according to the particle diameter which is a known experimental parameter. At each position $\vec{r}$ within the micrograph, we subtract the mean and divide by the the standard deviation.

$$\mu(\vec{r}) = \frac{1}{M_o} FT^{-1}\{FT(\mathbf{X})FT(\mathbf{M_o})^*\} \quad \text{(Equation 1)}$$

$$\sigma(\overrightarrow{r}) = \sqrt{\frac{1}{M_o} FT^{-1}\{FT(\mathbf{X}^2)FT(\mathbf{M_o})^*\} - \mu^2(\overrightarrow{r})} \quad \text{(Equation 2)}$$

Here, $\mathbf{X}$ is a matrix representing the micrograph, $\mathbf{M}_0$ is a circular mask based on the particle diameter, while $FT(\cdot)$ is the Fourier transform. This normalization procedure mitigates the influence of fluctuations in ice thickness, exposure, and other uncontrollable experimental variables, thereby enhancing the consistency of micrograph analysis.

Additionally, we adhere to a common procedure[21,30] where a Wiener filter is applied for denoising and Contrast Limited Adaptive Histogram Equalization (CLAHE) is used to enhance contrast by addressing non-uniform illumination and low contrast. Finally, guided filtering is applied using the CLAHE-enhanced image as a reference. This process selectively smooths the image while retaining important structural details, striking a balance between noise reduction and preservation of critical information. All images are resized to a shared dimension of 1024×1024. The steps above are summarized in Figure 1A.

Representation learning

Several families of self-supervised methods, such as Contrastive Learning,[63] Self-Distillation,[64] and Canonical Correlation,[65] rely on data augmentations that preserve the semantic content of data instances. Masked autoencoding, however, takes a completely different approach. We hypothesize that learning to reconstruct randomly masked patches of a micrograph can produce representations that capture particle-oriented local invariances without relying on augmentations. Consequently, the 1024×1024 resized micrographs are divided into 64×64 patches, generating 256 smaller images that cover the full micrograph. This approach addresses a key difference between cryo-EM and real-world images: micrographs lack the global spatial correlation seen in natural images. By focusing on smaller regions, the model emphasizes particles and preserves local context. Reducing the masking during training from 75% to 50% ensures that no particles are fully masked, while independent masking within each patch guarantees that masking is evenly spread across the micrograph. This prevents any single region from being completely masked or entirely visible, ensuring that the model receives balanced and representative input from all regions of the micrograph. Based on this, we propose the use of Masked Autoencoders (MAEs),[66] which have demonstrated the capability to learn representations encompassing a broad range of semantics relevant to downstream tasks, for representation learning on micrographs. During training, MAEs randomly mask a percentage of the input image, which is first divided into patches. The unmasked patches are processed by the encoder, which generates a latent representation for each patch. In the latent space, representations for the masked patches are added as empty placeholders. During the decoding step, the masked patches are predicted based on the latent information captured by the encoder from the unmasked patches. The learning objective of an MAE is to reconstruct the input image as faithfully as possible, achieved through the mean squared error (MSE) averaged per each image's patch: $MSE = \frac{1}{N}\sum_{i=1}^{N}(Y_i - \widehat{Y}_i)^2$. A perfect encoder should remain invariant across various levels of noise. Consequently, when clustering the representation space, distinct clusters are expected to emerge corresponding to different noise levels and distances from the particle centers. The micrograph representation step is illustrated in Figure 1B.

Implementation details

We use the ViT encoder[67] to learn the semantic information of micrograph patches at the embedding level. This is achieved through the Mean Squared Error (MSE) loss of the reconstructed patch provided by a ViT decoder:

$$MSE = \frac{1}{N}\sum_{i=1}^{N}(Y_i - \widehat{Y}_i)^2$$

Details of the architectural configurations, learning settings and image dimensions are provided in Table S5. To retain sufficient resolution at the latent embedding for the segmentation step, we patchify the original image before passing it through the model.

Inference

Dealing with various levels and noise fluctuations in micrographs complicates the accurate prediction of particles. Given an unseen micrograph, the inference process is performed in two stages **(i)** *clustering based on the learned representation space* and **(ii)** *smoothing and filtering of predictions*.

First, we address common high-frequency patterns and features of the non-particle regions shared across different micrographs by identifying these regions through the clustering derived from the latent representations of the training set. The choice of four clusters is the minimum required, as demonstrated in the ablations Figure 2B. A detailed explanation of clustering centers selection is provided in Subsection Ablations. After this initial filtration step, we address variations in noise levels across micrographs by applying clustering to the micrograph-specific latent representations. This approach allows dealing with different noise characteristics on the micrograph level. The clustering process is performed in three steps based on different number o cluster centers $k = i$, where $i = 3$, 4,5. At each clustering step, the cluster with the highest affinity to the previous step's particle cluster is selected. This process begins by defining the particle cluster using the latent representations of the training set through k-means clustering with four centers (computed once). A reference micrograph and its corresponding particle mask are used to identify the particle cluster. To segment each micrograph, we apply hierarchical clustering: first, using k-means with three clusters and selecting the one with the highest

overlap with the training-set-derived particle cluster, then repeating the process with four and five clusters, each time selecting the cluster with the highest similarity to the previous step's particle cluster. This process is illustrated in Figure 1C and displayed algorithmically in Algorithm 1 and Algorithm 2.

Algorithm 1. Train Set Clustering

Require: D: Set of feature representations from the training set
M_{ref}: Reference micrograph and its segmentation mask
1: Apply k-means clustering with 4 clusters on D.
2: Use M_{ref} to assign the particle cluster in the clustering of D.
3: **return** C_D: set of cluster centers (including the particle cluster c_p).

Algorithm 2. Micrograph-Specific Hierarchical Clustering

Require: M: Set of feature representations from the micrograph
C_D: Set of cluster centers from the training set clustering
c_p: Particle cluster of C_D
max_iters: Maximum number of clustering levels (default = 5)
1: $p \leftarrow 3$, $c_{p_3} \leftarrow c_p$
2: **for** $i = 3$ to max_iters **do**
3: Apply k-means clustering with i clusters on M
4: Set $c_{p_{i+1}}$ to the cluster with the greatest overlap with c_{p_i}
5: **end for**
6: **return** Indices of pixels that belong to the final particle cluster $c_{p_{max\_iters}}$

The initial predicted particle mask for the micrograph undergoes post-processing to extract the final predictions. First, convolution with a 3x3 kernel is applied to smooth the predictions, in order and fill occasional holes in the segmented particle masks. Then, bilinear interpolation restores the image to a higher resolution, for more accurate localization of the particles. Subsequently, a threshold is applied to the smoothed predictions to prune away low confidence segmentation masks. However, this threshold is contingent upon the unique experimental parameters and characteristics of each dataset and micrograph. Therefore, finding the optimal threshold for each micrograph is imperative. To accomplish this, predicted segmentation masks are computed using various thresholds within the [0, 1] range. The optimal threshold is determined by ensuring that the resulting segmentation mask aligns with the statistical properties of the training data, specifically ensuring that particles occupy approximately 4% of the micrograph. Finally, further post-processing is performed to filter out (i) neighboring predictions based on particle diameter (ii) filter predictions whose radius exceeds by a threshold the particle radius, and (iii) remove predictions at the borders of the micrographs, since at these position particles are usually partitioned. These steps are depicted in Figure 1D.

Experimental set-up

The rationale behind comparing with Topaz and crYOLO is obvious within the cryo-EM research community, where these two methods stand out as the most widely used deep learning tools for particle picking in Single Particle Analysis (SPA) of cryo-EM micrographs. Topaz and crYOLO also represent two distinct approaches: the former as an image classification model and the latter as an object detection model. Both utilize convolutional neural networks to learn features from the cryo-EM micrographs.

Four separate training procedures, each using a different EMPIAR dataset, were conducted for each of the three methods. The different EMPIAR datasets were provided by CryoPPP,[33] selected to maximize diversity. These datasets include EMPIAR: 10291, 10077, 10590, 10816, which have proteins of different diameters (160Å, 250Å, 237Å, and 180Å, respectively), each representing different protein type and function. The evaluation procedure was performed on the test sets of these four datasets and an additional set of 10 EMPIAR datasets, all annotated by CryoPPP. These datasets, include EMPIAR: 10028, 10081, 10096, 10240, 10406, 10289, 10737, 10059, 11183, 10017, have particle diameters ranging from 100Å to 300Å. For the results on the cell extracts of the EMPIAR: 10892 experiment, all three machine learning methods were trained on an extensive set of 20 EMPIAR datasets (EMPIAR: 10017, 10028, 10059, 10061, 10075, 10077, 10081, 10096, 10184, 10240, 10289, 10291, 10406, 10532, 10590, 10669, 10737, 10760, 10816, 11183) from cryoPPP[33] to ensure maximum generalization capabilities of the models. These datasets, comprising approximately 300 micrographs, were randomly divided into three subsets: 60% for training, 20% for validation, and 20% for testing. This diverse set of 20 EMPIAR datasets encompasses a wide range of protein types, functions, subcellular locations, organisms of origin, shapes, sizes, noise characteristics, and concentrations within the micrographs.

Particle picking can be perceived as an object detection problem, therefore we use the following common evaluation metrics in the literature[27,28]: (i) Intersection over Union (IoU) between annotated and predicted particles, using the particle diameter as the box size, (ii) recall, precision, and F1 score where true positives (TP) are only counted for predicted particles that uniquely overlap with ground truth particles by more than 60% of their surface area, while all other predicted particles are classified as false positives (FP).

Micrographs used for training all three methods underwent identical preprocessing steps as discussed in Micrograph preprocessing ensuring a fair comparison across the methodologies. For the training of Topaz, we employed the default ResNet8 model with the default training parameters, running for 400 epochs. To be aligned with the original work, the epoch yielding the highest area under the precision-recall curve on the validation set was selected. Micrographs were resized following the protocol outlined in.[23] For the training of the crYOLO, all micrographs were resized to the recommended resolution of 1024x1024, with anchor inputs based on the protein particle size in each dataset relative to resolution. The number of epochs and model checkpoints is adjusted in the code based on the reduction of validation set loss during training. cryo-EMMAE was trained for 400 epochs. Training cryo-EMMAE for 400 epochs takes about 2.5 hours per 100 micrographs. Inference takes around 130 seconds per 100 micrographs. Both computation times are reported on a system with an AMD Ryzen 7 5800X 8-Core Processor CPU and a single Nvidia RTX 4080 GPU. Further implementation details of cryo-EMMAE are presented in the Table S5.

3D reconstruction methodology

For the single-particle reconstruction analysis, eight test datasets (EMPIAR: 10028, 10081, 10017, 11183, 10289, 10406, 10077, 10291) were selected. MRC motion-corrected files provided by CryoPPP[33] were first imported into CryoSPARC, followed by patch CTF estimation for each micrograph. Picked particle coordinates from each method were saved in an STAR-formatted file, which was then imported into CryoSPARC for the 'Extract from Micrographs' job. The extracted box sizes were consistent with those used in the CryoPPP analysis. Two workflows were then employed. The first used all picked particles from the four methods for ab-initio reconstruction followed by homogeneous refinement. The second workflow included a single round of 2D classification and 2D class selection, followed by ab-initio reconstruction and homogeneous refinement. The 3D reconstructions of CryoPPP annotation particles are reported from the first workflow, as they are provided as a noise-free set. For datasets EMPIAR: 10081, 10017, 10289, 10291, the respective symmetries (C4, D2, C8, and C8) were imposed during homogeneous refinement, as suggested by their Electron Microscopy DataBank (EMDB) entries (experimental metadata "applied_symmetry" field). All other CryoSPARC jobs were executed using default parameters.

For the multi-particle reconstruction analysis, we first downloaded the multi-frame unaligned micrographs of entry EMPIAR: 10892. The authors of the original paper kindly provided the STAR file containing the picked particle coordinates. Based on these coordinates, we selected a subset of 854 micrographs from the initial 2808 unaligned micrographs. This subset included the top 300 micrographs with the highest particle abundance for each of the four reconstructed structures presented in the paper: (i) the pre-60S Ribosomal subunit, (ii) Fatty Acid Synthase (FAS), (iii) the E2 core of the Oxoglutarate Dehydrogenase complex (OGDHc), and (iv) the E2 core of the Pyruvate Dehydrogenase complex (PDHc). Due to overlaps in micrograph selection based on particle abundance, the total number of micrographs was smaller than 1200 (4×300). These 854 unaligned micrographs were first imported into cryoSPARC, followed by patch motion correction and patch CTF estimation. For each method, the picked particles were extracted using the largest box dimension (384px) of the four proteins. Since the dataset contained projections from multiple proteins and various sources of noise, two consecutive rounds of 2D classification (300 classes) and 2D class selection were conducted to clean the dataset. This step aimed to enrich abundant protein projections while removing low-abundance projections and noise. A final 2D classification (100 classes) and 2D class selection were then performed on the selected classes from the previous two rounds. For each of the four proteins, the corresponding projections were identified based on the ground truth particle classes and underwent ab-initio reconstruction and homogeneous refinement. Symmetries were imposed for FAS (D3), OGDHc (O), and PDHc (I), as reported in the paper. For the particle picks provided in the original paper, only the ab-initio reconstruction and homogeneous refinement steps were performed, with the respective symmetries applied during homogeneous refinement. All other cryoSPARC parameters were left at their default settings.

All the 3D reconstructed density maps presented at Figures 4, S5 and S6 are imaged with the use of ChimeraX v1.8-1.[62] Upon request to the lead contact, we can provide cryoSPARC jobs.

QUANTIFICATION AND STATISTICAL ANALYSIS

Data and results were analyzed using Python (version 3.10) and are presented as average values across different datasets. Principal component analysis (PCA) was performed using the PCA function from the scikit-learn module (version 1.2.2).

Cell Reports Methods, Volume *5*

Supplemental information

Self-supervised learning for generalizable particle picking in cryo-EM micrographs

Andreas Zamanos, Panagiotis Koromilas, Giorgos Bouritsas, Panagiotis L. Kastritis, and Yannis Panagakis

Supplemental information

Table S1: **Evaluation metrics for all test datasets, reported for each training dataset. Related to Table 1**. The metrics include: (i) Intersection over Union (IoU), measuring the overlap between ground truth and predicted particles; (ii) Recall, the ratio of correctly predicted particles, prediction counts as a true positive if IoU ≥ 0.6; (iii) Precision, the proportion of retrieved particles that are true positives; and (iv) F1 score, the harmonic mean of recall and precision. For cryo-EMMAE, the difference from the highest-performing or second-highest method is additionally reported for each entry and metric.

Trained on 10291

Test Sets	IoU			Recall			Precision			F1		
	Topaz	CrYOLO	EMMAE	Topaz	CrYOLO	EMMAE	Topaz	CrYOLO	EMMAE	Topaz	CrYOLO	EMMAE
10291	0.794	**0.819**	0.579 ↓0.24	**0.955**	0.928	0.597 ↓0.36	0.282	**0.475**	0.434 ↓0.04	0.435	**0.628**	0.503 ↓0.13
10077	0.039	0.566	**0.717** ↑0.15	0.021	0.604	**0.844** ↑0.24	0.219	0.224	**0.464** ↑0.24	0.038	0.327	**0.599** ↑0.27
10590	**0.684**	0.548	0.566 ↓0.12	**0.716**	0.550	0.581 ↓0.14	0.313	0.528	**0.562** ↑0.03	0.436	0.539	**0.571** ↑0.03
10816	0.000	0.010	**0.478** ↑0.47	0.000	0.009	**0.415** ↑0.41	0.000	**0.561**	0.234 ↓0.33	0.000	0.018	**0.299** ↑0.28
10028	0.000	0.558	**0.786** ↑0.23	0.000	0.619	**0.947** ↑0.33	0.000	0.276	**0.658** ↑0.38	0.000	0.382	**0.776** ↑0.39
10081	**0.791**	0.739	0.778 ↓0.01	**0.934**	0.844	0.863 ↓0.07	0.313	**0.673**	0.565 ↓0.11	0.469	**0.749**	0.683 ↓0.07
10096	0.210	0.011	**0.522** ↑0.31	0.135	0.004	**0.436** ↑0.30	0.237	0.161	**0.425** ↑0.19	0.172	0.008	**0.430** ↑0.26
10240	**0.565**	0.445	0.321 ↓0.24	**0.574**	0.428	0.299 ↓0.28	0.389	0.560	**0.563** - 0.00	0.464	**0.485**	0.391 ↓0.09
10406	0.017	0.639	**0.716** ↑0.08	0.008	0.655	**0.814** ↑0.16	0.094	0.187	**0.569** ↑0.38	0.015	0.291	**0.670** ↑0.38
10289	**0.735**	0.649	0.571 ↓0.16	**0.798**	0.664	0.553 ↓0.25	0.208	0.317	**0.361** ↑0.04	0.330	0.429	**0.437** ↑0.01
10737	**0.498**	0.476	0.444 ↓0.05	**0.490**	**0.490**	0.461 ↓0.03	0.205	**0.544**	0.459 ↓0.09	0.289	**0.516**	0.460 ↓0.06
10059	**0.677**	0.465	0.433 ↓0.24	**0.674**	0.436	0.340 ↓0.33	0.334	**0.620**	0.495 ↓0.13	0.447	**0.512**	0.403 ↓0.11
11183	0.489	0.309	**0.533** ↑0.04	0.486	0.293	**0.527** ↑0.04	0.173	**0.323**	0.287 ↓0.04	0.255	0.307	**0.372** ↑0.07
10017	0.454	0.018	**0.498** ↑0.04	0.457	0.006	**0.518** ↑0.06	0.569	0.205	**0.657** ↑0.09	0.507	0.012	**0.579** ↑0.07
Mean	0.425	0.447	**0.567** ↑0.12	0.446	0.467	**0.585** ↑0.12	0.238	0.404	**0.481** ↑0.08	0.276	0.372	**0.512** ↑0.14

Trained on 10077

Test Sets	IoU			Recall			Precision			F1		
	Topaz	CrYOLO	EMMAE	Topaz	CrYOLO	EMMAE	Topaz	CrYOLO	EMMAE	Topaz	CrYOLO	EMMAE
10291	**0.747**	0.233	0.601 ↓0.15	**0.851**	0.033	0.620 ↓0.23	0.289	0.057	**0.428** ↑0.14	**0.431**	0.042	0.506 ↑0.08
10077	**0.826**	0.784	0.708 ↓0.12	**0.983**	0.924	0.832 ↓0.15	0.348	**0.506**	0.46 ↓0.05	0.514	**0.654**	0.592 ↓0.06
10590	**0.664**	0.459	0.565 ↓0.01	**0.698**	0.368	0.577 ↓0.12	0.247	0.465	**0.553** ↑0.09	0.365	0.411	**0.565** ↑0.15
10816	0.489	0.000	**0.502** ↑0.01	0.389	0.000	**0.474** ↑0.09	0.147	0.000	**0.302** ↑0.16	**0.213**	0.000	0.369 ↑0.16
10028	**0.836**	0.815	0.788 ↓0.05	**0.995**	0.955	0.950 ↓0.05	0.408	0.630	**0.650** ↑0.02	0.579	0.759	**0.772** ↑0.01
10081	0.680	0.454	**0.779** ↑0.10	0.772	0.329	**0.865** ↑0.09	0.134	0.355	**0.550** ↑0.20	0.228	0.342	**0.672** ↑0.33
10096	0.018	0.025	**0.537** ↑0.51	0.003	0.002	**0.445** ↑0.44	0.010	0.036	**0.416** ↑0.38	0.005	0.004	**0.430** ↑0.43
10240	**0.615**	0.308	0.343 ↓0.27	**0.620**	0.206	0.320 ↓0.30	0.305	0.437	**0.564** ↑0.13	**0.409**	0.280	0.408 - 0.00
10406	0.775	**0.782**	0.717 ↓0.07	0.918	**0.930**	0.818 ↓0.11	0.440	**0.637**	0.575 ↓0.06	0.595	**0.756**	0.675 ↓0.08
10289	**0.667**	0.339	0.576 ↓0.09	**0.683**	0.140	0.559 ↓0.12	0.184	0.160	**0.357** ↑0.17	**0.290**	0.149	0.436 ↑0.15
10737	**0.596**	0.070	0.487 ↓0.11	**0.635**	0.043	0.502 ↓0.13	0.311	0.355	**0.463** ↑0.11	0.418	0.077	**0.482** ↑0.06
10059	**0.645**	0.196	0.432 ↓0.21	**0.612**	0.057	0.338 ↓0.27	0.413	0.191	**0.495** ↑0.08	**0.493**	0.088	0.402 ↓0.09
11183	**0.669**	0.014	0.533 ↓0.14	**0.703**	0.003	0.534 ↓0.17	0.210	0.054	**0.288** ↑0.08	0.323	0.006	**0.374** ↑0.05
10017	0.346	0.035	**0.486** ↑0.14	0.247	0.002	**0.505** ↑0.26	0.171	0.021	**0.645** ↑0.47	0.202	0.004	**0.566** ↑0.36
Mean	**0.612**	0.322	0.575 ↓0.04	**0.651**	0.285	0.596 ↓0.06	0.258	0.279	**0.482** ↑0.14	0.362	0.255	**0.518** ↑0.11

Trained on 10590

Test Sets	IoU			Recall			Precision			F1		
	Topaz	CrYOLO	EMMAE	Topaz	CrYOLO	EMMAE	Topaz	CrYOLO	EMMAE	Topaz	CrYOLO	EMMAE
10291	**0.714**	0.665	0.484 ↓0.23	**0.784**	0.717	0.502 ↓0.28	0.285	0.414	**0.449** ↑0.04	0.418	**0.525**	0.474 ↓0.05
10077	0.098	**0.690**	0.407 ↓0.28	0.116	**0.785**	0.477 ↓0.31	**0.644**	0.235	0.458 ↓0.19	0.197	0.362	**0.467** ↑0.11
10590	**0.788**	0.742	0.588 ↓0.20	**0.915**	0.834	0.599 ↓0.32	0.284	0.500	**0.543** ↑0.04	0.433	**0.625**	0.570 ↓0.06
10816	0.020	0.061	**0.127** ↑0.07	0.006	**0.059**	0.037 ↓0.02	0.023	**0.648**	0.036 ↓0.61	0.010	**0.108**	0.036 ↓0.07
10028	0.028	0.716	**0.787** ↑0.07	0.026	0.868	**0.939** ↑0.07	**0.714**	0.289	0.626 ↓0.09	0.050	0.434	**0.751** ↑0.32
10081	**0.829**	0.797	0.784 ↓0.04	**0.958**	0.921	0.870 ↓0.09	0.165	0.343	**0.551** ↑0.21	0.282	0.500	**0.675** ↑0.18
10096	**0.604**	0.326	0.533 ↓0.07	**0.543**	0.104	0.440 ↓0.10	0.171	0.131	**0.439** ↑0.27	0.260	0.116	**0.439** ↑0.18
10240	**0.740**	0.672	0.319 ↓0.42	**0.801**	0.701	0.297 ↓0.50	0.294	0.476	**0.55** ↑0.07	0.430	**0.567**	0.386 ↓0.18
10406	0.369	0.667	**0.717** ↑0.05	0.404	0.689	**0.813** ↑0.12	**0.568**	0.271	0.557 ↓0.01	0.472	0.389	**0.661** ↑0.19
10289	**0.705**	0.700	0.527 ↓0.18	0.742	**0.743**	0.509 ↓0.23	0.230	0.294	**0.351** ↑0.06	0.351	**0.421**	0.415 ↓0.01
10737	**0.539**	0.400	0.472 ↓0.07	**0.562**	0.415	0.485 ↓0.08	0.352	**0.549**	0.455 ↑0.09	0.433	**0.473**	0.470 - 0.00
10059	0.488	**0.530**	0.214 ↓0.32	0.430	**0.456**	0.170 ↓0.29	**0.459**	0.531	**0.513** ↓0.02	0.444	**0.491**	0.255 ↓0.24
11183	0.044	**0.276**	0.115 ↓0.16	0.034	**0.239**	0.043 ↓0.20	0.082	**0.314**	0.072 ↓0.24	0.048	**0.271**	0.054 ↓0.22
10017	**0.762**	0.468	0.506 ↓0.26	**0.847**	0.278	0.521 ↓0.33	0.242	0.274	**0.626** ↑0.35	0.376	0.276	**0.569** ↑0.19
Mean	0.481	**0.551**	0.470 ↓0.08	0.512	**0.558**	0.479 ↓0.08	0.322	0.376	**0.444** ↑0.07	0.300	0.397	**0.444** ↑0.05

Trained on 10816

Test Sets	IoU			Recall			Precision			F1		
	Topaz	CrYOLO	EMMAE	Topaz	CrYOLO	EMMAE	Topaz	CrYOLO	EMMAE	Topaz	CrYOLO	EMMAE
10291	0.498	**0.732**	0.571 ↓0.16	0.231	**0.799**	0.584 ↓0.22	0.040	0.306	**0.427** ↑0.12	0.068	0.443	**0.493** ↑0.05
10077	0.587	0.629	**0.712** ↑0.08	0.435	0.622	**0.833** ↑0.21	0.050	0.102	**0.455** ↑0.35	0.090	0.175	**0.589** ↑0.41
10590	0.452	**0.729**	0.577 ↓0.15	0.170	**0.795**	0.588 ↓0.21	0.026	0.272	**0.548** ↑0.28	0.045	0.405	**0.567** ↑0.16
10816	**0.798**	0.734	0.511 ↓0.29	**0.931**	0.800	0.511 ↓0.42	0.134	0.363	**0.443** ↑0.08	0.234	**0.499**	0.475 ↓0.02
10028	0.517	0.660	**0.781** ↑0.12	0.289	0.735	**0.943** ↑0.21	0.030	0.193	**0.642** ↑0.45	0.054	0.306	**0.764** ↑0.46
10081	0.426	**0.801**	0.747 ↓0.05	0.139	**0.909**	0.828 ↓0.08	0.011	0.211	**0.554** ↑0.34	0.020	0.342	**0.664** ↑0.32
10096	0.358	0.378	**0.495** ↑0.12	0.128	0.205	**0.406** ↑0.20	0.029	0.221	**0.461** ↑0.24	0.047	0.213	**0.432** ↑0.22
10240	0.500	**0.683**	0.338 ↓0.35	0.269	**0.708**	0.311 ↓0.40	0.058	0.353	**0.545** ↑0.19	0.095	**0.471**	0.396 ↓0.08
10406	0.513	0.586	**0.701** ↑0.12	0.216	0.455	**0.792** ↑0.34	0.031	0.125	**0.557** ↑0.43	0.054	0.196	**0.654** ↑0.46
10289	0.449	**0.724**	0.572 ↓0.15	0.205	**0.765**	0.554 ↓0.21	0.038	0.216	**0.354** ↑0.14	0.064	0.337	**0.432** ↑0.10
10737	0.612	**0.733**	0.351 ↓0.38	0.565	**0.791**	0.364 ↓0.43	0.078	0.233	**0.453** ↑0.22	0.137	0.360	**0.404** ↑0.04
10059	0.491	**0.602**	0.416 ↓0.19	0.222	**0.532**	0.324 ↓0.21	0.087	0.415	**0.496** ↑0.08	0.125	**0.466**	0.392 ↓0.07
11183	0.526	**0.614**	0.493 ↓0.12	0.397	**0.609**	0.482 ↓0.13	0.080	0.204	**0.297** ↑0.09	0.133	0.306	**0.368** ↑0.06
10017	0.488	0.409	**0.489** - 0.00	0.280	0.300	**0.508** ↑0.21	0.054	0.344	**0.649** ↑0.31	0.091	0.320	**0.570** ↑0.25
Mean	0.515	**0.644**	0.554 ↓0.09	0.320	**0.645**	0.573 ↓0.07	0.053	0.254	**0.492** ↑0.24	0.090	0.346	**0.514** ↑0.17

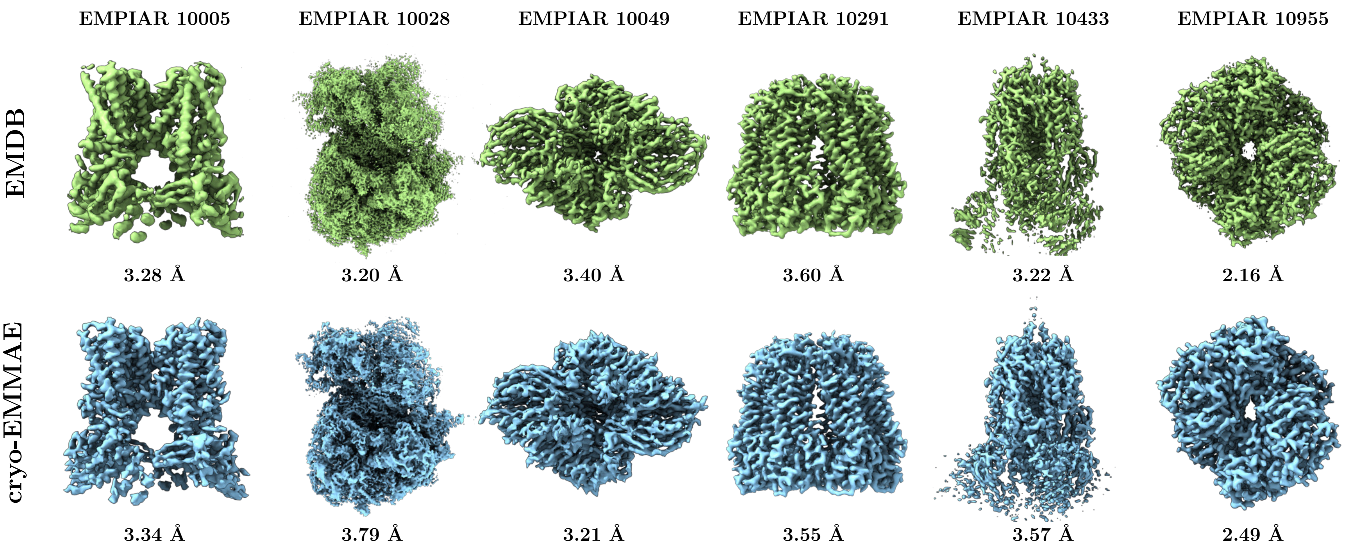

Figure S1: **Visualization of 3D reconstructed density maps from the published volumes in EMDB and cryo-EMMAE across six different EMPIAR datasets. Related to Real Case Studies subsection of the main manuscript.** EMDB maps are shown in green, while those generated using cryo-EMMAE are shown in blue. Resolutions are also reported under each volume. Maps produced with cryo-EMMAE underwent only one round of 2D classification and were not subjected to any post-processing of the 3D reconstructed map.

Table S2: **Comparison of 3D reconstruction resolutions and the number of particles used between the EMPIAR published datasets and the cryo-EMMAE particle picker. Related to Real Case Studies subsection of the main manuscript.** Results are reported after one round of 2D classification and selection, with mean values also provided.

Symmetry	EMPIAR	3D Reconstruction Resolution		Number of Particles	
		EMPIAR	cryo-EMMAE	EMPIAR	cryo-EMMAE
C4	10005	**3.28Å**	3.34Å ↑0.06Å	35,645	44,215
C1	10028	**3.20Å**	3.79Å ↑0.59Å	105,247	134,330
C2	10049	3.40Å	**3.21Å** ↓0.19Å	108,544	138,153
C8	10291	3.60Å	**3.55Å** ↓0.05Å	54,536	49,975
C3	10433	**3.22Å**	3.45Å ↑0.23Å	54,395	55,733
D2	10955	**2.16Å**	2.49Å ↑0.33Å	88,731	40,418
	Mean	**3.14Å**	3.30Å ↑0.16Å	74,516	77,137

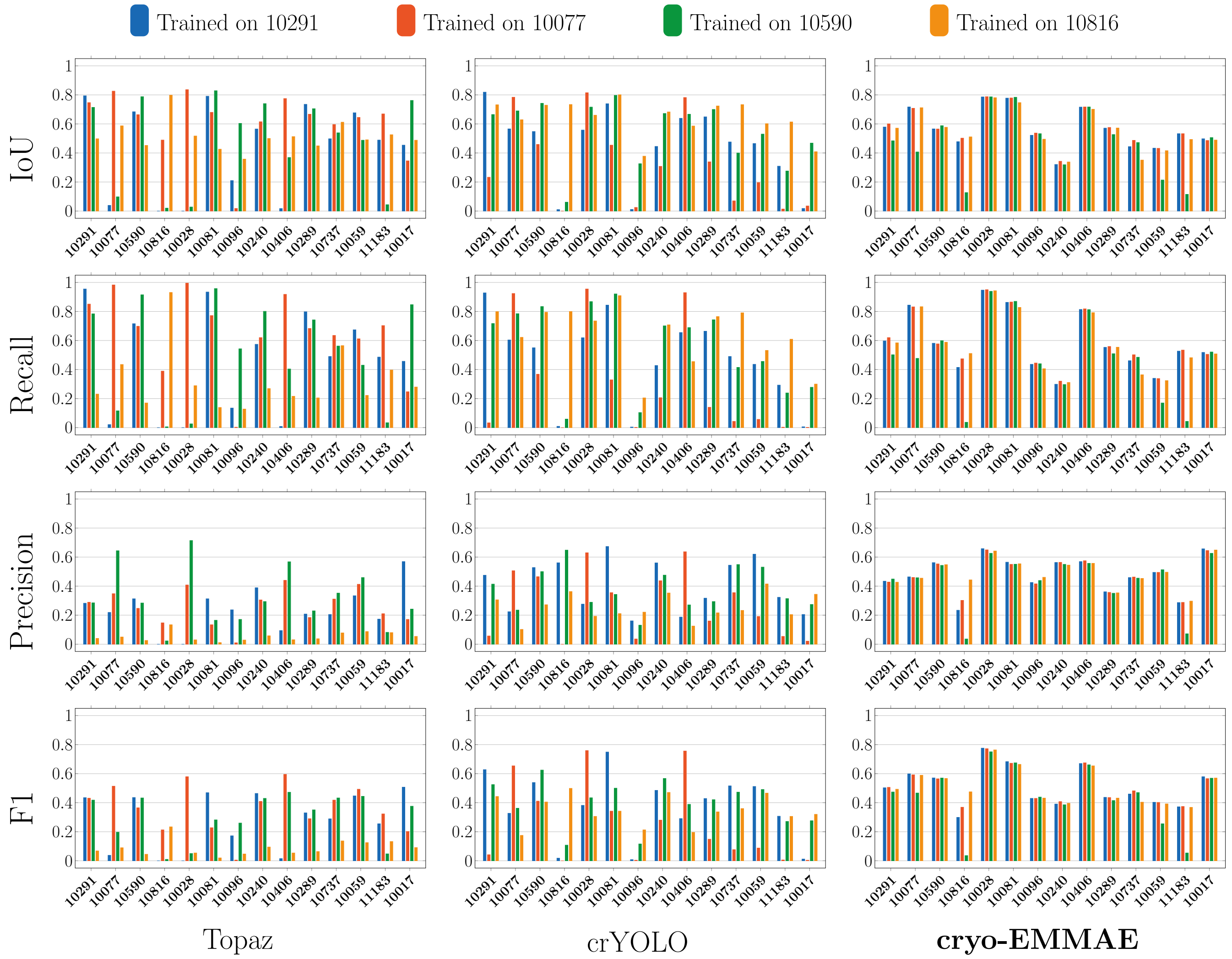

Figure S2: **Performance bar plots. Related to Table 1.** Bar plots depict the comparative performance of Topaz, crYOLO, and cryo-EMMAE across four key metrics (IoU, Recall, Precision, F1), trained on distinct datasets (10291, 10077, 10590, 10816), distinguished by color. Evaluation is conducted on 14 datasets, illustrated on the horizontal axis.

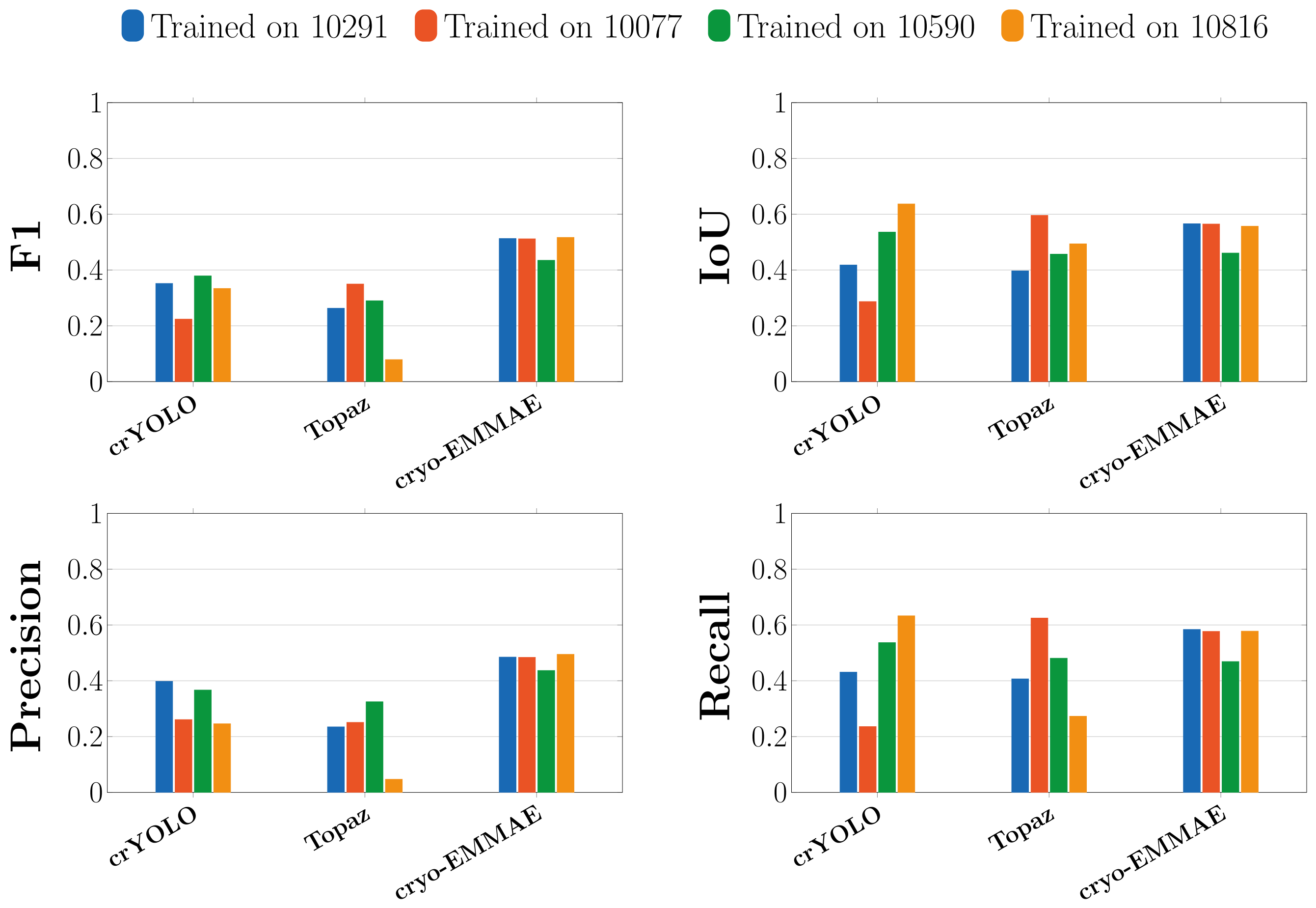

Figure S3: **Overall performance bar plots. Related to Table 1.** Bar plots show the comparative performance of Topaz, crYOLO, and cryo-EMMAE across four key metrics (IoU, Recall, Precision, F1), with each method trained on different datasets (10291, 10077, 10590, 10816), represented by distinct colors. The metrics are averaged over the 13 test datasets.

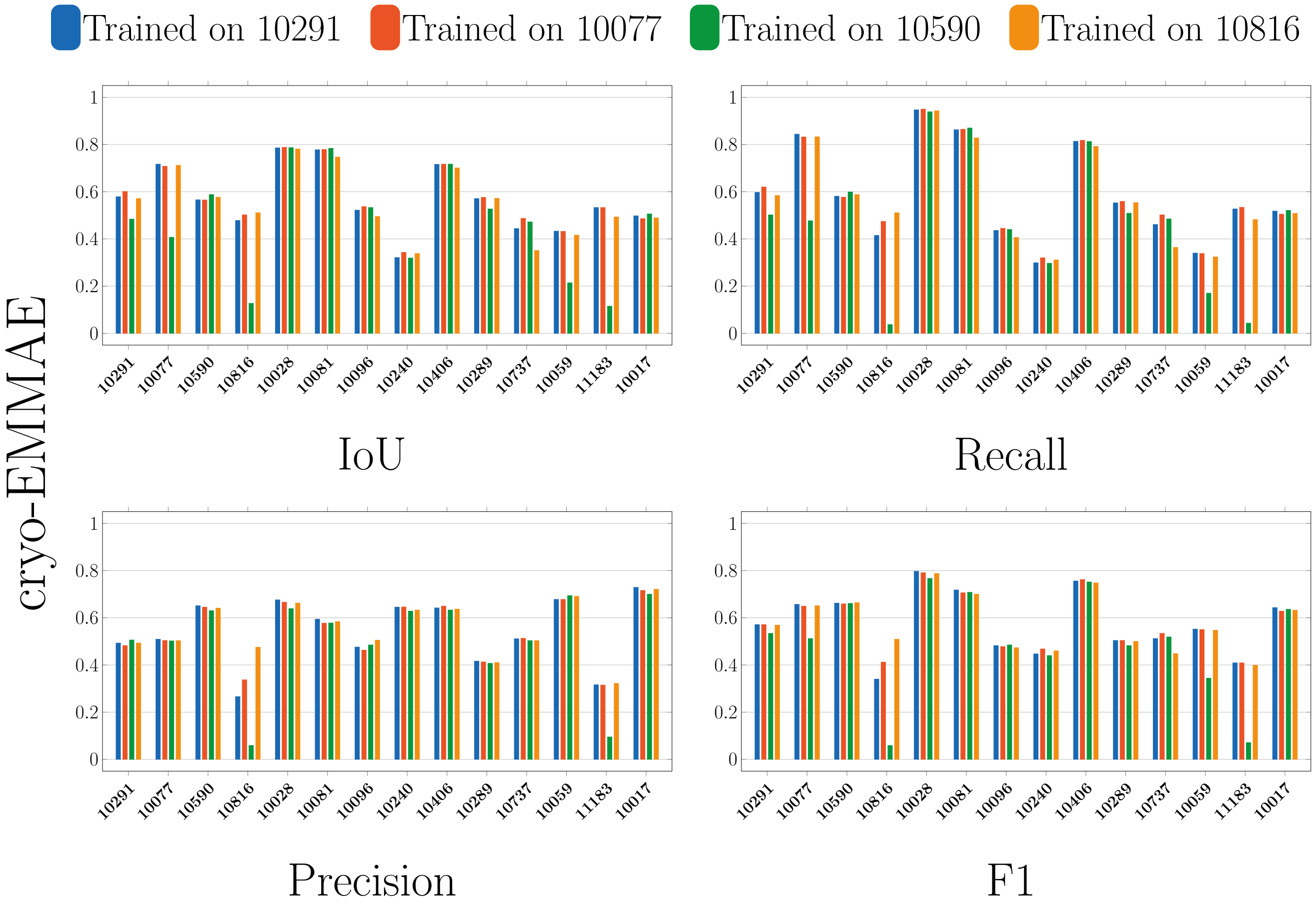

Figure S4: **Performance bar plots for 0.5 IoU threshold. Related to Table 1.** Performance of Cryo-EMMAE for IoU threshold of 0.5 across four metrics (IoU, Recall, Precision, F1), trained on distinct datasets (10291, 10077, 10590, 10816). Evaluation conducted on 14 datasets.

Table S3: **Mean distances of latent representations for particle and background regions. Related to Figure 3.** Mean distances are computed within Micrograph A, within Micrograph B, and across Micrographs A and B. Each block reports distances between region types (Particles or Background).

Micrograph	Region	Particles	Background
A (within)	Particles	3.08	3.32
	Background	3.32	3.16
B (within)	Particles	3.35	3.45
	Background	3.45	3.33
A $\rightarrow$ B (cross)	Particles	3.63	3.53
	Background	3.94	3.65

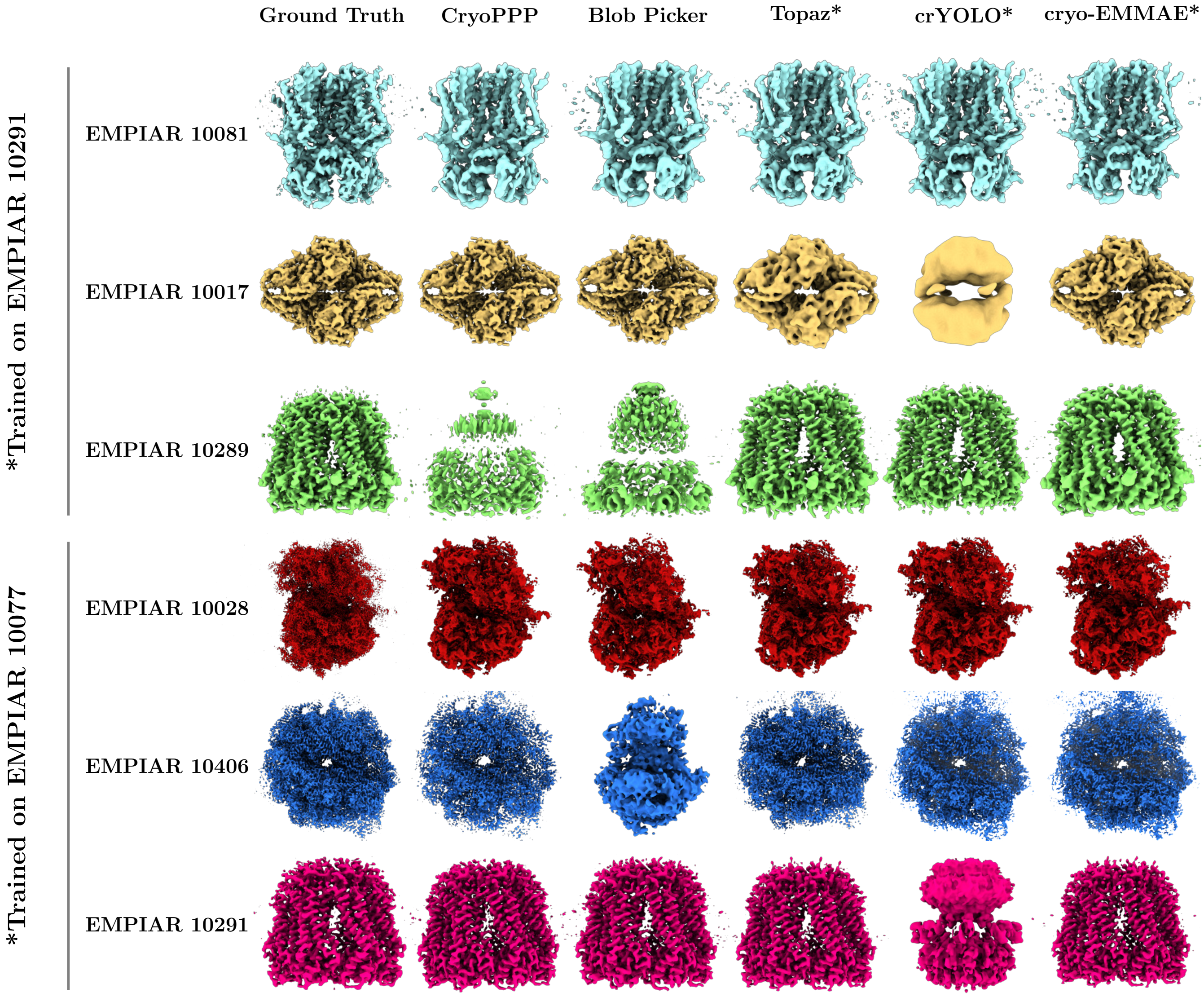

Figure S5: **Visualization of reconstructed density maps of ground truths and the four methods. Related to Table 2.** Each EMPIAR dataset is represented by a different color for clarity. For all columns except Ground Truth, reconstructed volumes were generated from a subset of approximately 300 micrographs, except for EMPIAR 10017, where only 84 micrographs were used. For the learning-based methods (Topaz, crYOLO, and cryo-EMMAE), the first three datasets were reconstructed using models trained on EMPIAR 10291, while the last three used models trained on EMPIAR 10077. The volumes reconstructed by the four methods (Blob Picker, Topaz, crYOLO, and cryo-EMMAE) include the 2D classification step, whereas the reconstructions of CryoPPP (annotation) particles do not include 2D classification.

Table S4: **Number of picked particles per reconstruction. Related to Table 2.** Number of picked perticles for eight different test datasets across four methods: Blob Picker, Topaz, crYOLO, and cryo-EMMAE. The last three methods were trained on datasets 10291 and 10077. The number of picked particles is reported both with and without 2D classification, with the percentage of retained particles included for the latter. Entries that did not successfully reconstruct the expected density map are highlighted in gray. Averaging is computed only for the correctly reconstructed entries.

Test Set	**Trained on 10291**								
	Without 2D Classification				With 2D Classification				
	Blob Picker	Topaz	crYOLO	cryo-EMMAE	Blob Picker	Topaz	crYOLO	cryo-EMMAE	CryoPPP GT
10028	32,102	0	19,272	35,997	29,067 (90.6%)	0 (-%)	17,147 (89.0%)	32,293 (89.7%)	25,050
10081	80,829	49,723	77,459	57,805	38,325 (47.4%)	38,019 (76.5%)	40,725 (52.6%)	36,828 (63.7%)	39,315
10017	50,160	4,057	268	37,552	45,445 (90.6%)	3,329 (82.1%)	232 (86.6%)	31,926 (85.0%)	49,178
11183	152,700	43,964	27,747	146,172	68,100 (44.6%)	32,624 (74.2%)	11,010 (39.7%)	54,218 (37.1%)	79,736
10289	93,384	119,992	85,657	91,127	48,178 (51.6%)	55,396 (46.2%)	41,608 (48.6%)	34,292 (37.6%)	61,455
10406	33,763	0	32,447	32,654	28,782 (85.3%)	0 (-%)	24,968 (86.1%)	27,922 (85.5%)	24,669
10077	70,990	0	28,929	56,808	22,546 (31.8%)	0 (-%)	19,597 (67.7%)	32,889 (57.9%)	31,834
Mean	73,418	31,105	38,826	65,445	40,063 (63.1%)	18,481 (69.7%)	22,184 (67.2%)	35,767 (65.2%)	44,462
	**Trained on 10077**								
Test Set	Without 2D Classification				With 2D Classification				
	Blob Picker	Topaz	crYOLO	cryo-EMMAE	Blob Picker	Topaz	crYOLO	cryo-EMMAE	CryoPPP GT
10028	32,102	33,016	32,022	36,589	29,067 (90.6%)	29,127 (88.2%)	29,127 (91.0%)	31,844 (87.0%)	25,050
10081	80,829	39,852	7,945	58,933	38,325 (47.4%)	15,237 (38.2%)	4,165 (52.4%)	38,674 (65.6%)	39,315
10017	50,160	518	426	37,742	45,445 (90.6%)	283 (54.6%)	175 (41.1%)	34,696 (91.9%)	49,178
11183	152,700	102,490	1,123	144,618	68,100 (44.6%)	60,733 (59.3%)	1,123 (100.0%)	54,077 (37.4%)	79,736
10289	93,384	92,627	18,597	92,627	48,178 (51.6%)	33,837 (36.5%)	10,267 (55.2%)	31,167 (33.7%)	61,455
10406	33,763	30,916	29,746	32,775	28,782 (85.3%)	29,793 (96.4%)	28,854 (97.0%)	28,804 (87.9%)	24,669
10291	172,240	73,632	20,153	135,795	105,731 (61.4%)	50,063 (68.0%)	14,253 (70.7%)	81,099 (59.7%)	99,765
Mean	87,883	53,293	15,716	77,011	51,947 (67.3%)	31,296 (63.0%)	12,566 (72.5%)	42,909 (66.2%)	54,167

Table S5: **Architecture parameters of ViT encoder/decoder and configuration parameters for cryo-EMMAE. Related to Figure 1.**

**Configuration**	**ViT Encoder**	**ViT Decoder**
Embedding Dimensions	192	128
Transformer Layers	14	7
Attention Heads	1	8
MLP Ratio	2.0	2.0
**General Configuration**		
Initial Image Shape	1024×1024	
Image's Number of Patches	256	
ViT Input Image Shape	64×64	
ViT Number of Patches	16	
ViT Patch Shape	4×4	
Optimizer	AdamW	
Base Learning Rate	1.0×10^{-3}	
Weight Decay	1.0×10^{-5}	
Optimizer Momentum	$\beta_1, \beta_2 = 0.9, 0.999$	
Batch Size	128	
Training Images	$\approx$ 46,000	
Mask Ratio	0.5	

Table S6: **Comparison of 3D reconstruction resolutions and the number of particles used between the EMPIAR published datasets and machine learning-based particle pickers. Related to Single Particle 3D Reconstructions subsection of the main manuscript.** Results are reported after one round of 2D classification and selection, with mean values also provided. The only exception is EMPIAR 10049 for Topaz, where two rounds of 2D classification were required.

Symmetry	EMPIAR	**3D Reconstruction Resolution**				**Number of Particles**			
		EMPIAR	Topaz	crYOLO	cryo-EMMAE	EMPIAR	Topaz	crYOLO	cryo-EMMAE
C1	10028	**3.20Å**	3.76Å	3.79Å	3.79Å ↑0.59Å	105,247	138,735	125,947	134,330
C2	10049	3.40Å	3.25Å	3.25Å	**3.21Å** ↓0.04Å	108,544	168,389	180,900	138,153
C3	10433	**3.22Å**	3.43Å	3.54Å	3.45Å ↑0.23Å	54,395	59,872	53,011	55,733
D2	10955	**2.16Å**	2.50Å	3.04Å	2.49Å ↑0.33Å	88,731	91,049	9,903	40,418
	Mean	**3.00Å**	3.24Å	3.40Å	3.24Å ↑0.24Å	89,229	114,511	92,440	92.158

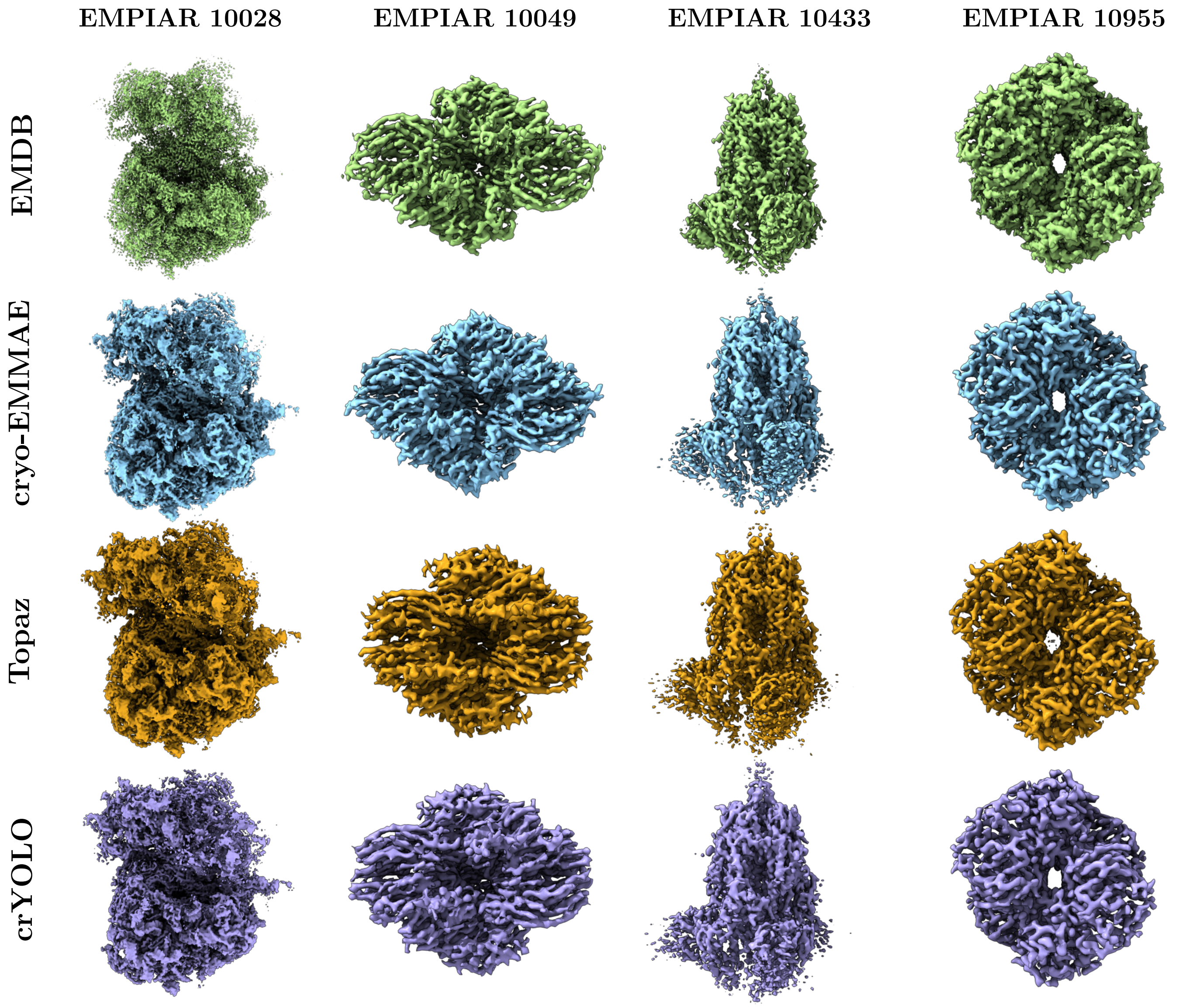

Figure S6: **Visualization of 3D reconstructed density maps from the published volumes in EMDB, cryo-EMMAE, Topaz, and crYOLO evaluating across four different EMPIAR datasets. Related to Single Particle 3D Reconstructions subsection of the main manuscript.** The three machine learning methods have been **trained on 20 EMPIAR datasets**. EMDB maps are shown in green, those generated using cryo-EMMAE in blue, Topaz in gold, and crYOLO in purple. Maps produced by the machine learning methods underwent only one round of 2D classification and were not subjected to any post-processing of the 3D reconstructed map. The only exception is EMPIAR 10049 for Topaz, where two rounds of 2D classification were required. The density threshold is selected using the ChimeraX sdlevel command, which determines the threshold based on standard deviations (SD) from the mean for each 3D density map.

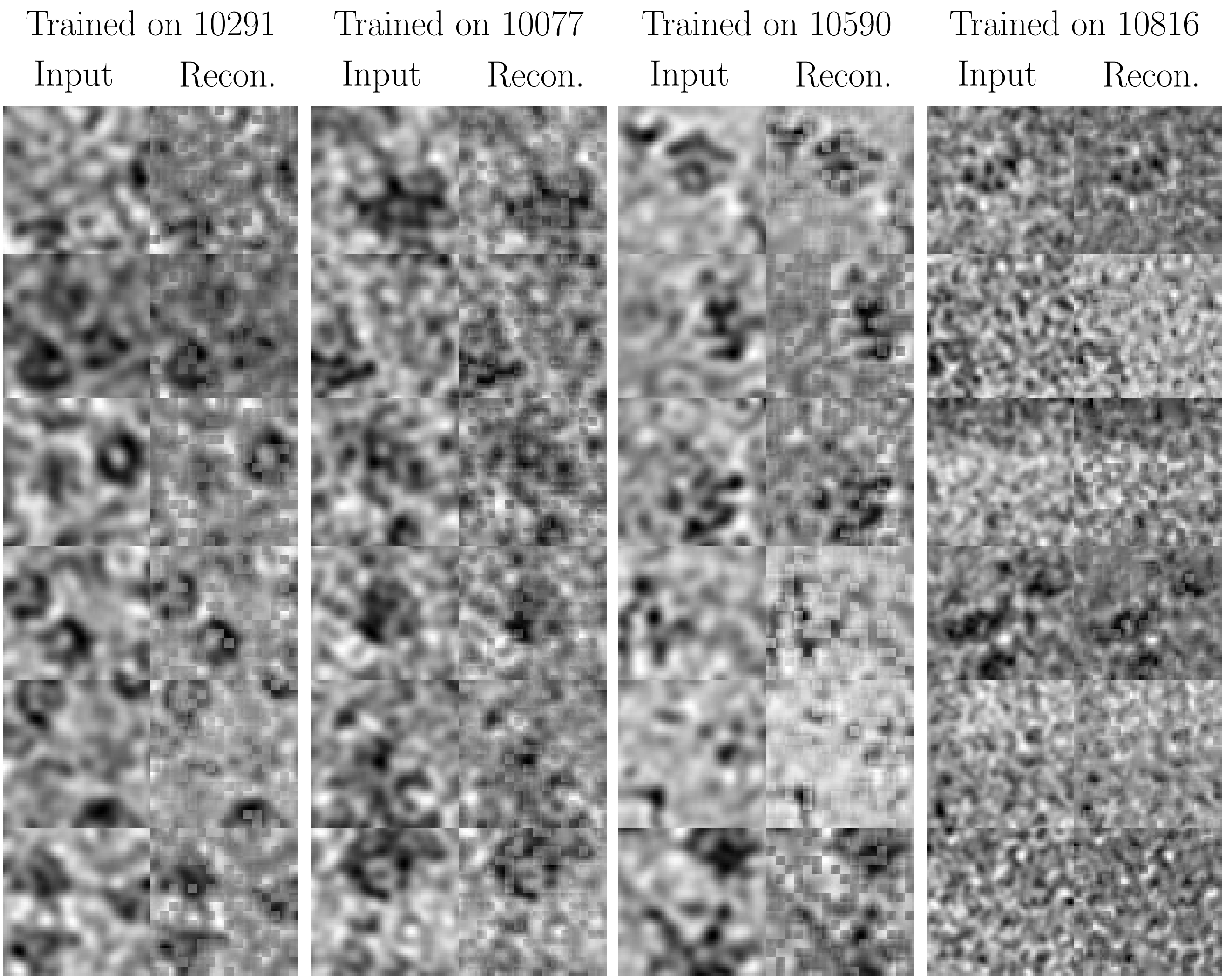

Figure S7: **Examples of input and reconstructed images from Cryo-EMMAE with a mask ratio of 0.5. Related to Figure 1.** Models trained independently on four different EMPIAR datasets: 10291, 10077, 10590, and 10816.
